# Molecular fluctuations as a ruler of force-induced protein conformations

**DOI:** 10.1021/acs.nanolett.1c00051

**Published:** 2021-03-25

**Authors:** Andrew Stannard, Marc Mora, Amy E.M. Beedle, Marta Castro-López, Stephanie Board, Sergi Garcia-Manyes

**Affiliations:** 1 Department of Physics, Randall Centre for Cell and Molecular Biophysics and London Centre for Nanotechnology, King’s College London, Strand, WC2R 2LS London, United Kingdom; 2 The Francis Crick Institute, 1 Midland Road, London NW1 1AT, London, UK

**Keywords:** protein nanomechanics, single molecule magnetic tweezers, protein fluctuations, energy landscape, protein folding, protein stiffness

## Abstract

Molecular fluctuations directly reflect the underlying energy landscape. Variance analysis examines protein dynamics in several biochemistry-driven approaches, yet measurement of probe-independent fluctuations in proteins exposed to mechanical forces remains only accessible through steered molecular dynamics simulations. Using single molecule magnetic tweezers, here we conduct variance analysis to show that individual unfolding and refolding transitions occurring in dynamic equilibrium in a single protein under force are hallmarked by a change in the protein’s end-to-end fluctuations, revealing a change in protein stiffness. By unfolding and refolding three structurally distinct proteins under a wide range of constant forces, we demonstrate that the associated change in protein compliance to reach force-induced thermodynamically-stable states scales with the protein’s contour length, in agreement with the sequence-independent freely-jointed chain model of polymer physics. Our findings will help elucidate the conformational dynamics of proteins exposed to mechanical force at high resolution, of central importance in mechanosensing and mechanotransduction.

Mechanical unfolding of proteins regulates a variety of biological processes, encompassing muscle elasticity^1^, focal adhesion mechanosensing^2, 3^ and nuclear translocation^4^. Whether working as monomers or in form of polyproteins^5^, mechanical unfolding provides an efficient shock absorbing strategy that works as a safety mechanism to preserve the mechanical integrity of adhesive and mechanotransducing protein complexes^6^. Several strategies —including the protein’s intrinsic topology, the introduction of point mutations, ligand binding, the pulling direction or the dynamic rupture/reformation of covalent bonds— have been reported to govern the mechanical resistance of proteins in their folded form^7^. Once unfolded, mechanically stretched proteins exhibit previously cryptic sites to the solvent, which become suddenly available to protein binding or post-translational modifications, ultimately affecting protein elasticity^8^. Given the emerging evidence of conformation-dependent functionality^9^, it is enticing to experimentally capture the mechanical properties of the different protein forms, collectively defining the rich conformational repertoire of proteins under force. For example, the so far elusive measurement of protein stiffness could provide an additional tool, further to the unfolding force and contour length increment measurement^7^, to characterise the diverse conformational dynamics of proteins, using the spatial magnitude of the fluctuations of each force-induced equilibrium conformation as its structural fingerprint.

Single molecule nanomechanical experiments have provided a detailed in-vitro insight of the molecular determinants governing the conformational dynamics of proteins under force^8, 10^. Collectively, these measurements have concentrated on providing an extensive characterization of the mechanical stability of the native state of proteins^11^, and on capturing the kinetics and out-of-equilibrium transition paths underpinning individual unfolding and refolding trajectories^12^. By contrast, a thorough experimental description of the extended states —often perceived as a single, featureless, compliant conformation —has comparatively received much less attention. This largely stems from the lack of experimental approaches able to capture the evolution of protein fluctuations with force, which emerge naturally from a general and rather simplified picture of the folding energy landscape that defines the behaviour of an entropic ideal polymer under force (predicted by models of protein elasticity^13^ e.g. the freely-jointed chain (FJC) or worm-like chain (WLC)) added to an enthalpic, short-range attractive contribution (e.g. Morse or Lennard-Jones potential)^14^. In the absence of force, proteins populate this latter energy basin, which defines the native state of the protein (Fig. 1). The application of a constant force tilts the 1D energy landscape and creates a new energy minimum that corresponds to the protein’s unfolded state. Increasing the stretching force lowers the energy of the unfolded well and displaces its position toward increasingly extended conformations, as predicted by the FJC/WLC model. Consequently, the width of the unfolded minimum decreases when increasing the stretching force. Hence, the measurement of the length fluctuations as a function of force should provide an accurate and direct measurement of protein stiffness, which is a direct, natural consequence of the curvature of the energy well (Fig. 1). Despite its theoretical simplicity, a systematic experimental quantification of the dependency of protein stiffness with protein extension has evaded characterization, mainly due to the technical inability to capture the natural fluctuation modes of the protein when exposed to the wide range of stretching forces that fingerprint its entire conformational space, encompassing both the native and unfolded conformations.

Here we employ single molecule magnetic tweezers^15–18^, which enable (*i*) capturing of the unfolding/refolding trajectories of the same single protein over extended (hour-long) periods of time with high stability^16^, (*ii*) under a wide range of constant forces (2-100 pN), (*iii*) in passive clamp mode (hence avoiding potential masking of the protein fluctuations due to the active electronic feedback^19^), (*iv*) in the absence of long tethers (which necessarily add additional inherent fluctuations to the protein construct^20^), and, most importantly, (*v*) in an experimental set-up where the measured stiffness is dominated by the intrinsic stiffness of the protein-of-interest (and not the pulling device, such as e.g. the AFM cantilever^21–23^, Fig. S1 and SI text) to capture the change in compliance upon mechanical unfolding/refolding of individual proteins, in both monomeric and polymeric forms. Our experiments enable direct observation of the change in protein stiffness upon mechanical unfolding and over a continuum range of force-induced conformations in thermodynamic equilibrium, and demonstrate that molecular fluctuations are a reliable reporter of force-induced protein conformations.

## Results

Using single molecule magnetic tweezers, we monitored the unfolding and refolding trajectories of an individual protein L (PL) monomer bracketed between two mechanically rigid Ig32 titin domains either side. The resulting (Ig32)_2_-PL-(Ig32)_2_ polyprotein is covalently attached to the glass substrate through a Halo-Tag on the N-terminus, and to the paramagnetic bead via biotin-streptavidin binding on the C-terminus (Fig. 2A and Methods). Using a force quench protocol, we first applied a constant force of 38 pN, which elicited the unfolding of the individual PL monomer, fingerprinted by a step elongation of the protein by Δ<*x*> = 16.8 nm ^16^. We note that, at this force, we did not observe the unfolding of the Ig32 domains, ensuring that the folding dynamics of the PL monomer are not masked by the dynamics of the stiff Ig32 molecular handles (Fig. S2). After 60 seconds, the force was quenched down to 8.1 pN –the force, *F*_0.5_, at which PL exhibits a 50% folded probability– and held constant for 60 minutes. During this period of time, we observed the stochastic unfolding and refolding of the PL monomer (hallmarked by an average step-change in extension of Δ<*x*> = 10.3 ± 0.3 nm) occurring with approximate equal rates^16^, Fig. 2B. The protein was finally stretched back again at 38 pN to ensure that, over this long pulling time, the protein had not misfolded.

To experimentally measure changes in the mechanical properties of PL each time the monomer hops between the folded and unfolded states, we first consider that the measured stiffness, *k*_M_, of the entire series construct (combining the stiffness of PL, *k*_P_, and the rest of the construct, *k*_C_) is given by 1/*k*_M_ = 1/*k*_P_ + 1/*k*_C_. As such, upon un/folding, the change in measured stiffness does not correspond to the change in PL stiffness (Δ*k*_M_ ≠ Δ*k*_P_). If instead we consider compliance –the inverse of stiffness, *c* = 1/*k*– we have the simple situation where *c*_M_ = *c*_P_ + *c*_C_ and, crucially, the change in measured compliance is the change in PL compliance (Δ*c*_M_ = Δ*c*_P_). In the simple case of the classic Hookean spring, equipartition theory implies that constant (force-independent) compliance is given by (1)

(1) $$c=\beta \sigma^{2}$$

where *β* = 1/*k*_B_ *T* is the inverse of thermal energy, and *σ* ^2^ = <*x* ^2^> – <*x*>^2^ is the variance in end-to-end length, *x*. The trivial consequence is that the length fluctuations are force independent. By contrast, in the case of an entropic spring (e.g. in the FJC and WLC models) where the average length <*x*> is non-linear with force, compliance is force-dependent since *c* ≡ d<*x*>/d*F*. Even in this case, Eq. 1 still holds true (SI text), implying that fluctuations are force-dependent, being lower at higher forces, and can be used to determine compliance of non-linear springs such as proteins. Combining these two arguments, we see that the change in PL compliance can be found from the change in measured variance, Δ*c*_P_ = *β*Δ*σ*_M_^*2*^.

**FIG. 1:**
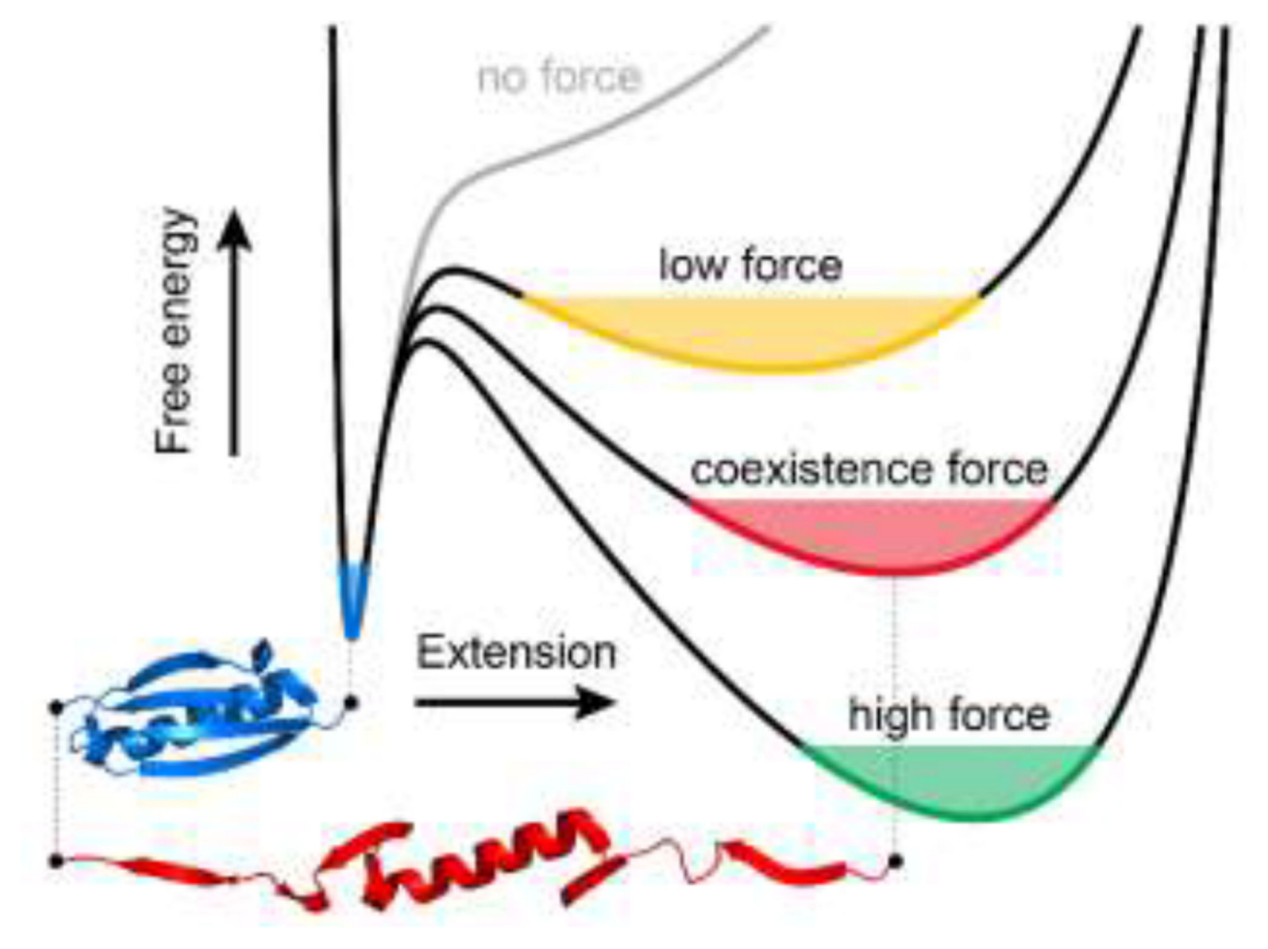
The effect of force on the free energy landscape of protein (un)folding. Using the example of protein L, in the absence of force (grey line), there is one minimum, corresponding to the folded state (blue). When a low force is applied, a broad local minimum corresponding to an unfolded state emerges (yellow). At a certain, coexistence force (red), the protein will equally likely be found in folded and unfolded states. As force increases, the unfolded minimum becomes narrower, eventually becoming the global minimum (green).

**FIG. 2:**
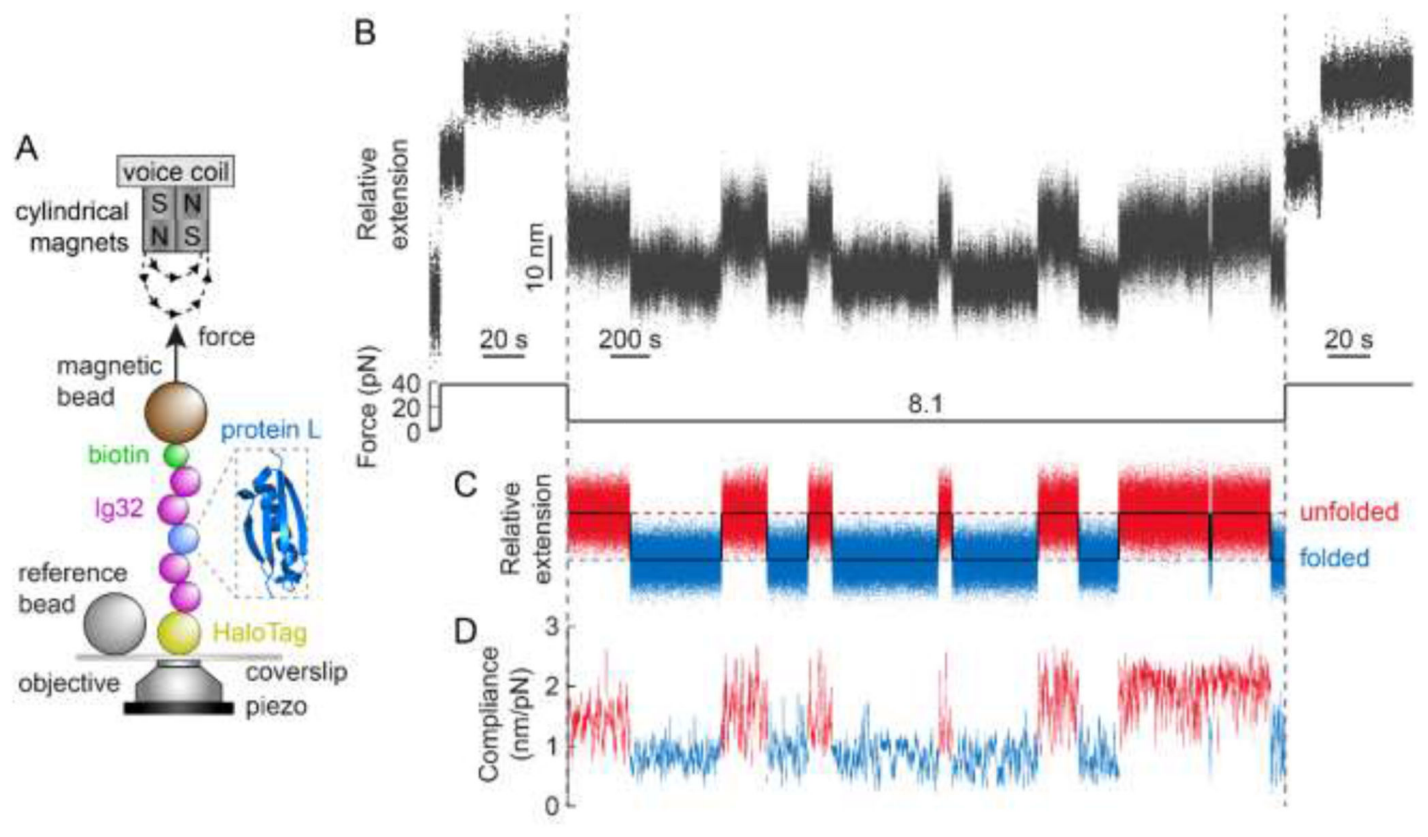
A step-wise change in protein compliance fingerprints mechanical unfolding/refolding of a single PL monomer under force. **(A)** Schematic illustration of PL (PDB: 1HZ6) construct being stretched in a magnetic tweezers set-up. **(B)** Raw extension-time measurement of a protein L monomer hopping in equilibrium between the folded and unfolded states. The (Ig32)_2_-PL-(Ig32)_2_ construct is initially pulled at 38 pN, which extends and unfolds the PL monomer. After 60 s, the force is quenched down to 8.1 pN for 60 minutes. During this time the PL monomer stochastically hops between the folded and unfolded states in steps of Δ<*x*> = 10.3 ± 0.3 nm. A final pulse at a high force (38 pN) value triggers the (re)unfolding of the PL monomer. **(C)** Result of flattening the protein extension data during the 8.1pN pulse to remove slow drift effects, clearly highlighting 13 individual (un)folding transitions. **(D)** Compliance of (C) (calculated over a 3-second moving window), showing step-wise changes (Δ*c* = 0.86 ± 0.09 nm/pN) concomitant to the PL individual unfolding and refolding events.

We next processed the hopping trajectory of Fig. 2B by flattening individual sections to remove the effects of slow timescale drift of the magnetic bead relative to the reference bead (<10 nm/hour average). Applying Eq. 1 to the resulting trajectory (Fig. 2C) enables direct measurement of the protein’s compliance (Fig. 2D) corresponding to the unfolded and folded states before and after each individual (un)folding transition^24^ (SI text and Fig. S3). By averaging the compliance for both, the natively-folded (blue) and unfolded (red) states underpinning the 13 individual transitions in Fig. 2, we obtained an increase in protein compliance Δ*c* = 0.86 ± 0.09 nm/pN upon PL unfolding (see also Fig. S4). Of note, the fluctuations underpinning the (un)folding dynamics of the PL monomer are independent of the rest of the polyprotein construct as observed when replacing the stiff Ig32 handles by the inextensible Spy0128 protein (Fig. S5). Conceptually, this increase in compliance corresponds to the transition of the protein, at a constant force (8.1 pN), from the narrow basin defining the native state to the much-broader well defining the unfolded state of the protein (Fig. 1).

**FIG. 3:**
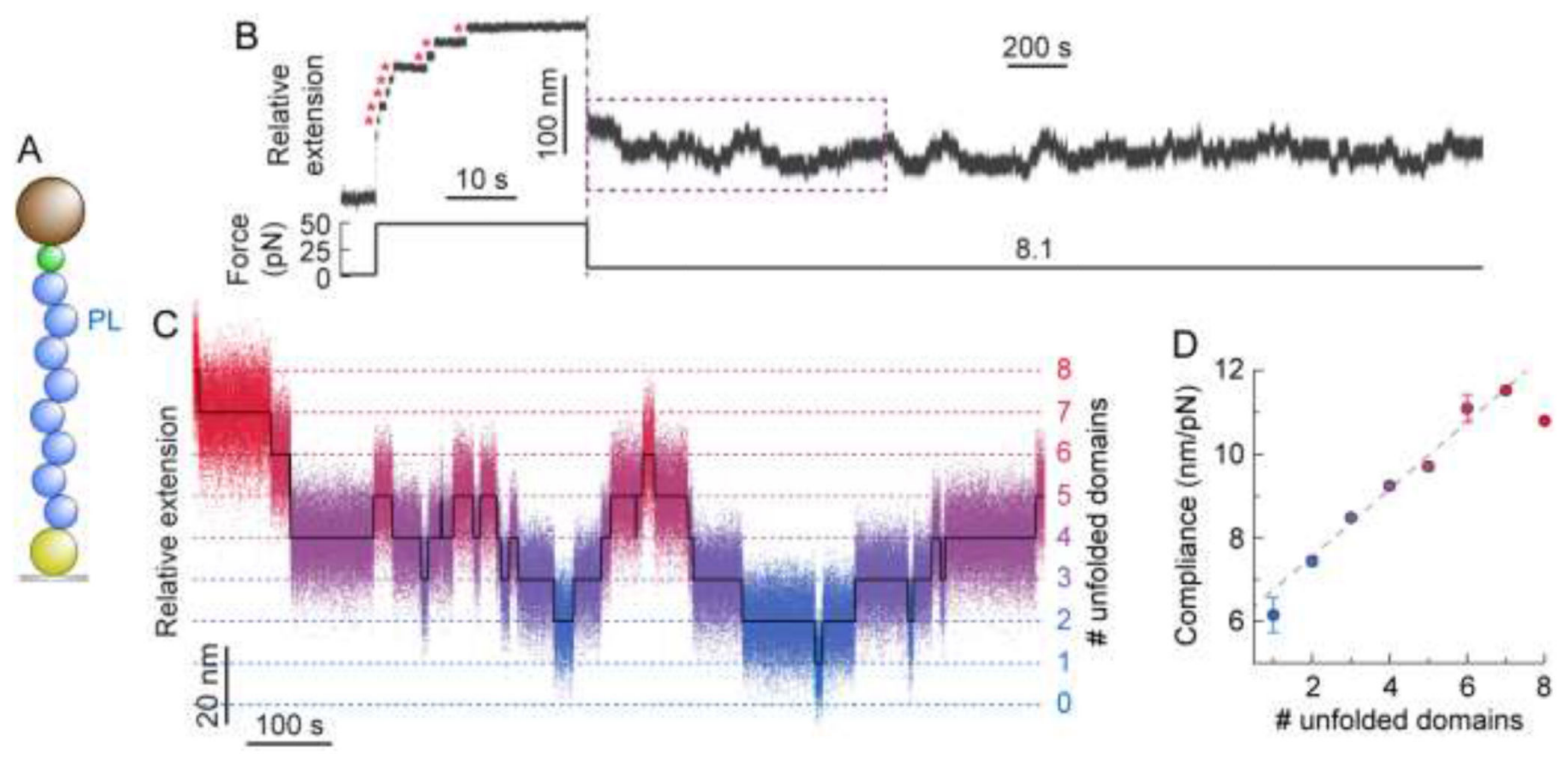
The change in compliance in a PL_8_ polyprotein is additive and recapitulates that of an individual domain. **(A)** Illustration of the PL_8_ construct being pulled in the MT set-up. **(B)** Typical (un)folding hopping trajectory of PL_8_ under force. Following a force-quench protocol, a high force pulse (49 pN) is first applied, to trigger the unfolding of each individual domain within the polyprotein chain (red asterisks), hallmarked by changes in extension of 17.2 nm. The force was subsequently dropped down to 8.1 pN (resulting in an initial decrease in extension) and held for 50 minutes. During this period of time, the PL_8_ polyprotein dynamically hops between well-defined states separated by Δ<*x*> = 10.7 ± 0.1 nm. **(C)** Segment flattening and colour-based assignment of the number of unfolded domains allows for clearer visualisation of the step-wise hopping dynamics. **(D)** The average compliance (*n* = 131 (un)folding events) can be calculated for a given number of unfolded domains. Linear fit (weighted by the total observation time at each level) to the data yields a gradient of 0.81 ± 0.15 (*R* ^2^ = 0.983), corresponding to the change in compliance of an individual domain.

Having obtained a measurable change in protein compliance upon mechanical unfolding of an individual protein monomer, we conjectured that, in the more complex case of a polyprotein (Fig. 3A), we might be able to single out the (un)folding of each individual domain by directly measuring the fluctuation variance of the entire construct at a given time and under constant force. To test this hypothesis, we repeated our force-quench protocol on a PL_8_ polyprotein (Fig. 3B). The long quench pulse at 8.1 pN exhibited complex dynamics, underpinning the stochastic unfolding/refolding transitions of each distinct protein monomer forming the polyprotein chain (Fig. 3C). Measuring the variance of the overall fluctuations of the polyprotein construct at each state (number of unfolded domains) level revealed a linear correlation between the construct’s compliance and the number of unfolded domains (Fig. 3D and Fig S6). Notably, the slope of the trend (Δ*c*/domain = 0.81 ± 0.15 nm/pN) is in very good agreement with that obtained for the monomeric protein (Fig. 2D). These results further highlight that the different monomers forming a polyprotein chain fold independently, implying that the folding dynamics of an individual monomer is independent of the composition of the rest of the multidomain construct^25, 26^. Most importantly, our results reveal that the dynamic change in compliance of a multi-protein construct can be used as a direct proxy to identify the number of folded (and unfolded) modules within an actively folding homopolyprotein at a given time.

**FIG. 4:**
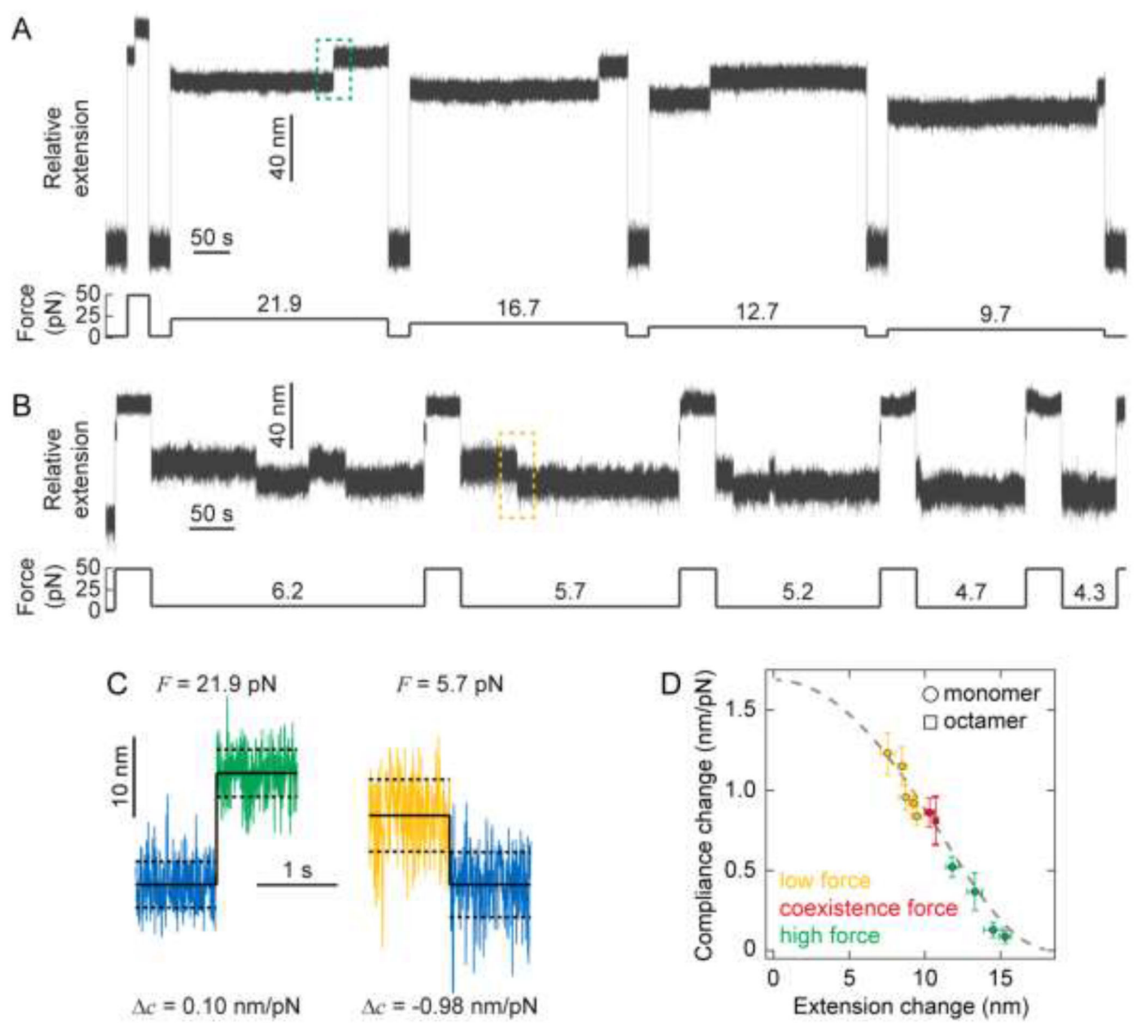
Correlating force-dependent extension and compliance changes upon mechanical (un)folding through the FJC model of polymer elasticity. **(A)** Cyclic unfolding and refolding trajectories allow probing the change in protein stiffness upon PL unfolding at varying high forces. Using a force quench protocol, the protein is initially held at a seemingly low force (1.8 pN) for 30 seconds to ensure correct refolding. The force is then increased to a higher constant force value in each cycle (namely 21.9, 16.7, 12.7, and 9.7 pN) for 5 minutes, which triggers in each case the unfolding of the PL monomer (marked by a step-wise increase in length). (**B**) Similar cyclic force-quench protocol to capture the change in protein compliance upon mechanical refolding at low forces. Before each cycle, the force is first kept low (1.8 pN) for 30 seconds to reach the protein’s native state. Subsequently, in each cycle the protein is unfolded at high force (49.2 pN) for 40 seconds, before the force is successively quenched at low forces, namely 6.2 pN (5 min), 5.7 pN (4 min), 5.2 pN (3 min), 4.7 pN (2 min) and 4.3 pN (1 min), respectively. When held at such low force values, the protein stochastically refolds back to the native state, as evidenced by the step-wise reduction in length. While the folded state dominates at these forces, some hopping back to an unfolded state is observed. **(C)** Representative unfolding (left) and folding (right) events occurring at high (21.9 pN) and low (5.7 pN) forces respectively (corresponding to the green dashed box in (A) and the yellow dashed box in (B), respectively). High force unfolding is hallmarked by a large increase in average extension but a small increase in compliance, conversely low force folding is hallmarked by a small decrease in average extension but a large decrease in compliance. (**D)** Plot of (absolute) compliance change against (absolute) extension change combining low (yellow), hopping (red), and high (green) force experiments, clearly showing that, upon PL un/folding, an inverse correlation between its changes in extension and compliance is measured. Data points for monomers (circles) correspond to the mean ± s.e. of 9–17 events occurring at each force, data for the PL_8_ octamer (square) is taken from Fig. 3D. Combined, this data show very good agreement with the FJC model (grey dashed line) with Kuhn length *b* = 1.1 nm, *k*_*B*_ *T* = 4.04 pN nm, and Δ*L* = 18.6 nm.

In the equilibrium experiments reported thus far, the pulling force was held constant to acquire sufficient statistics of the two protein states as the protein hopped between the unfolded/folded conformations. To systematically study the effect of force on protein compliance, we expanded the range of probed forces by conducting successive unfolding/refolding cycles on the same single protein monomer (Fig. 2A), where the unfolding/refolding force was varied in the range 4.3-22 pN (Figs. 4A&B). We then measured the relative change in the fluctuation variance (and the related change in protein compliance) for each particular force (Fig. 4C&D). These results clearly demonstrate that, when PL is mechanically unfolded, the greater the change in average extension (due to higher force being applied), the lesser the change in compliance. Finally, our resulting data on the extension-dependent change in compliance was compared to the prediction by the FJC model – which has analytical solutions for Δ<*x*> and Δ*c* (Supplementary Information). Our experimental data, including both the monomer and polymer measurements, quantitatively agree with the FJC model (Fig. 4D), confirming that the change in compliance (ultimately measured through the protein’s end-to-end length fluctuations) is reduced as the pulling forces are increased, suggesting that the findings might be general and independent of the studied protein since the FJC has no consideration for protein structure.

**FIG. 5:**
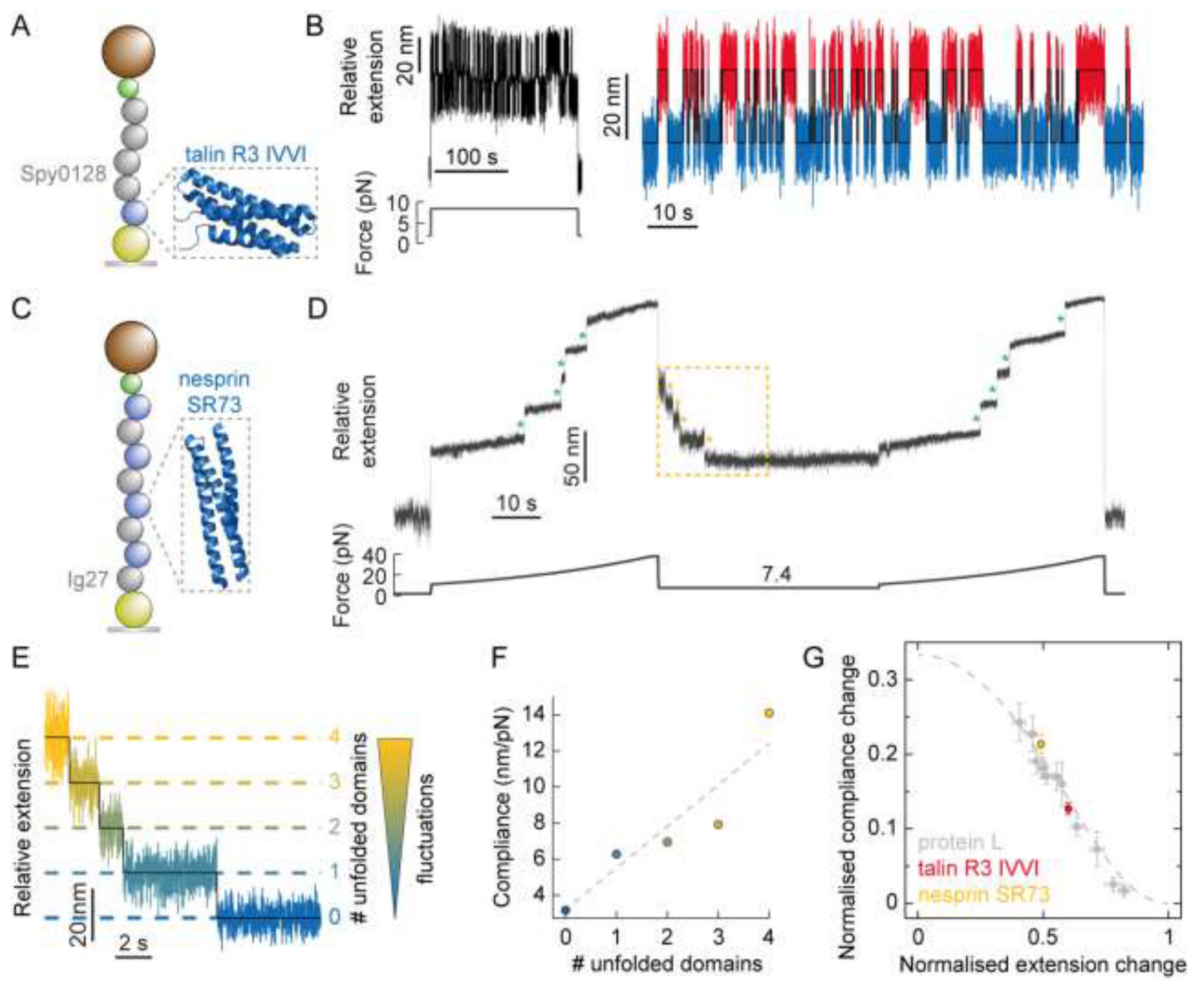
Direct observation of the change in end-to-end fluctuations upon individual talin and nesprin unfolding/refolding events. **(A)** Illustration of the [(Spy0128)_2_-TalinR3_IVVI_] construct being pulled in the MT set-up (PDB: 2L7A). **(B)** (Left) Raw, unfiltered extension-time data corresponding to the stretching of individual talin R3 IVVI monomer at a constant force of 8.35 pN for 200 seconds. During this time, the protein monomer undergoes multiple unfolding and refolding transitions. (Right) Zoom in on the hopping section, showing 84 consecutive unfolding(red)/refolding(blue) transitions concomitant to an average change in extension of Δ<*x*> = 22.2 ± 0.2 nm, and an average change in compliance between the folded and unfolded states of Δ*c* = 1.24 ± 0.14 nm/pN. Averaging over 5 such cycles gives Δ<*x*> = 22.2 ± 0.1 nm and Δ*c* = 1.28 ± 0.08 nm/pN. **(C)** Illustration of the [Ig27-SR73]_4_ construct being pulled in the MT set-up (lacking crystal structure, here we display the similar SR16 (PDB: 1U4Q). **(D)** Typical individual (unfiltered) folding trajectory under a force quench. The force is first increased from 10.6 pN to 37.5 pN over the course of 30 s (corresponding to the linear movement of magnet position by 1.4 mm over this time), resulting in the sequential step-wise unfolding of the four SR73 domains of nesprin (green asterisks). Note that, within this force range, the mechanically rigid Ig27 domains in the construct do not unfold. The force is then quenched down to 7.4 pN for 30 seconds, triggering the collapse and refolding of nesprin. At such a low (<*F*_0.5_) force value, refolding is relatively slow, such that the staircase-like pattern that mirrors that of unfolding can be captured. The force is subsequently ramped up again (test pulse) back to 37.5 pN, to probe that all the individual SR73 domains (green asterisks) had effectively refolded during the force-quench. (**E**) Zoom (yellow square) on the initial folding section of (B), where the four SR73 domains of nesprin refold sequentially, marked by a step-wise reduction of the protein extension by Δ<*x*> = 17.1 ± 0.5 nm. The refolding of each individual domain occurs concomitant to a reduction in the end-to-end fluctuations of the protein construct. **(F)** Plotting the compliance against the number of unfolded domains measured in (C) allows calculation of the change in compliance per domain, Δ*c*/domain = 2.30 ± 0.59 nm/pN. Averaging over 10 such folding trajectories with four SR73 nesprin unfolded domains gives Δ<*x*> = 16.8 ± 0.2 nm and Δ*c*/domain = 1.99 ± 0.12 nm/pN. **(G)** Plotting normalised compliance change (Δ*c*/*β* *b*Δ*L*) against normalised extension change (Δ*x*/Δ*L*) crucially reveals that the observed behaviour is protein independent, showing quantitative agreement with a master curve of the FJC model.

A closer look to the analytical expressions obtained from the FJC model (Supplementary Information) reveal that both the force-dependent extension change, and the force-dependent compliance change, are predicted to be linear with contour length increment. To experimentally test this hypothesis, we studied the change in compliance as an individual monomer IVVI mutant of the R3 rod domain of talin – a focal adhesion sensor^3^ featuring alpha-helix topology (Fig. 5A) and an associated increment in contour length of Δ*L* ≈ 37 nm^27, 28^ (which is significantly larger than that of PL, Δ*L* = 18.6 nm^29^) – hops in equilibrium between the folded and unfolded states at a constant force of 8.35 pN^30^ (Fig. 5B). Analysis of the hopping trajectories (following the same procedure used in Fig. 2B,C) reveals a significantly larger average increase in protein compliance Δ*c* = 1.28 ± 0.08 nm/pN upon unfolding.

A particular characteristics of the R3 domain of talin is its fast (un)folding/dynamics and its steep force-dependency for both the unfolding and refolding reactions^30^, which implies that at forces only slightly higher [lower] than the hopping (*F*_0.5_) force, the lifetime of the folded [unfolded] state becomes too short-lived to make a thorough measurement of compliance through the length variance (Fig. S7). To circumvent this limitation, we moved to conducting folding measurements on the spectrin 73 repeat (SR73) of nesprin, a nuclear envelope mechanosensor protein that has been suggested to play a key role in propagating extracellular mechanical forces to the cell nucleus^31^ likely through mechanical unfolding^32^ and which exhibits a comparable contour length increment (Δ*L* = 34.2 nm, Fig. S8C). Stretching an individual [Ig27-SR73]_4_ polyprotein (Fig. 5C) in a force-ramp (initial pulse) elicits the stepwise unfolding of the 4 individual SR73 nesprin domains, concomitant to an average increase in the protein length of Δ<*x*>= 28.6 nm (Fig. 5D, Fig. S8A,C). After complete unfolding of the nesprin SR73 domains with force ramped up to 38 pN—at this force we did not observe any signature of unfolding of the Ig27 marker (Fig. S2)—, the force was then quenched down to a lower value of 7.4 pN to trigger protein folding. We chose this force value because, according to the force-dependent folding probability that we measured for the nesprin SR73 domains (Fig. S8A,B), nesprin folds slow enough to directly capture the independent folding transitions corresponding to the sequential refolding of each individual SR73 monomer. Figure 5D shows how, akin to PL^16^ and to several domains of titin^33^, the previously mechanically uncharacterised nesprin mechanosensor effectively refolds against a constant force (7.4 pN), marked by the sequential stepwise shrinking (Δ<*x*> = 16.8 ± 0.2 nm) of the protein construct. To elucidate whether the sequential stepwise recoil of the protein (Fig. 5E) is mirrored by a step decrease in the end-to-end fluctuations of the polyprotein as each individual monomer refolds, we conducted variance analysis of the refolding trajectory over time and found that, on average the folding of each individual domain results in a concomitant change of the construct’s compliance by Δ*c* = 1.99 ± 0.12 nm/pN (Fig. 5F and Fig. S8D,E). Normalization of the measured change in compliance with the normalised change in extension for proteins with markedly different contour lengths and over a wide range of forces collapses data onto a FJC master curve, further demonstrating that the change in protein compliance is force- and length-dependent (through the FJC relationship) but—at least for the different proteins tested here—protein(sequence)- independent (Fig. 5G).

## Discussion

Analysis of molecular fluctuations has enabled capturing the conformational dynamics of proteins using a number of complementary experimental techniques, such as nuclear magnetic resonance (NMR) and small-angle X-ray scattering (SAXS)^34, 35^. At the single molecule level, sm-FRET has provided invaluable insight into the broad conformational heterogeneity and dynamics of the unfolded state of proteins, as well as of intrinsically disordered motifs^36^. More recently, interferometric scattering microscopy (iSCAT) has shown its ability to capture conformational transitions, provided they are associated to a change in the protein’s center of mass^37^. However, none of these experimental approaches can capture the effect of mechanical load on the conformational dynamics of individual proteins.

When denatured by force, proteins undergo a conformational change that brings the mechanically stiff, natively folded form into a stretched, compliant and unfolded conformation that cannot be populated in classical biochemical experiments using temperature or chemical denaturants^7, 38^. Single molecule nanomechanical techniques, mostly using AFM in the high force-regime^7^ and optical tweezers to probe proteins with lower mechanical stabilities^39–41^, have enabled sampling the regions of the protein free energy landscape that cannot be accessed through any other experimental means, and have rationalized the effect of force on modulating the conformational dynamics of an individual protein during its folding journey.

In this vein, earlier experiments using force-clamp AFM showed that, during collapse from highly-extended states, proteins exhibit increased end-to-end length fluctuations, reminiscent of the temporal occupancy of locally broad energy minima, before reaching their natively folded length ^14, 25, 42, 43^. Conformational heterogeneity was also captured at the molten globule^44^ and native states^45^ levels, however these experiments required using the mechanical stability of each conformation as its indirect structural fingerprint. Nonetheless, the high applied forces, the intrinsic stiffness (and mass) of the AFM probe and its associated force uncertainty (and decreased diffusion coefficient^21^), in addition to the active PID feedback, have precluded direct capture of the intrinsic fluctuations and underlying stiffness defining each force-dependent protein conformation. Our experiments using magnetic tweezers (where the stiffness of the protein is not masked by the pulling probe) demonstrate that the end-to-end fluctuations of a single protein under force can be used as a direct reporter of its precise conformation and folding status, hence introducing an additional experimentally measurable dimension to the sudden changes in protein length accompanying protein unfolding/refolding events. Noteworthy, these measurements are independent of the mechanical stability or the fold of the studied protein. The approach presented here does not require knowledge of the exact protein conformation of the protein before and after a transition, or even whether that transition has fleetingly-occupied intermediate states, and it is applicable to any conformational change that involves a change in the protein’s contour length. This methodology may be of particular relevance to identify time-dependent events occurring without obvious changes in the protein’s end-to-end length, such as ligand/drug binding^46^ and post-translational modifications^47–49^ that place dihedral angle constrictions to the force-induced protein conformation^50, 51^. For example, binding of vinculin to unfolded talin^52^ and α-catenin^17^ is a central step in cellular mechanotransduction. Importantly, all these functionalities can be finely regulated by the pulling force through precise modulation of protein stiffness and conformation, as revealed, with atomic detail, by steered molecular dynamics (SMD) simulations^53^. It will be enticing to compare, for distinct proteins, how our experimental results agree with the equilibrium fluctuations measured for equivalent force-induced protein conformations in SMD simulations^53^. More broadly, our experiments focusing on the size of natural molecular fluctuations defining the continuum force-induced thermodynamic equilibrium states complement recent elegant single molecule experiments concentrating on the non-equilibrium transition paths^12, 54, 55^ between those defined protein states.

## Methods

### Polyprotein engineering

The different protein constructs were designed, cloned and purified as reported elsewhere^16^. Details for each construct are described in detail in the Supplementary Information section.

### Single-molecule magnetic tweezers experiments

Sample preparation and the single-molecule magnetic tweezers setup are based on those reported elsewhere^16^, with slight variations detailed in the the Supplementary Information section.

## Supplementary Material

Supplementary information
