# Supplementary material for: Molecular fluctuations as a ruler of force-induced protein conformations
Source: Nano Lett. Author manuscript; Available in PMC 2021 May 4. (PMC7610714; doi:10.1021/acs.nanolett.1c00051)
Supplement: Supplementary information [file EMS122678-supplement-Supplementary_information.docx]

**Supplementary Online Material for:**

**Molecular fluctuations as a ruler of force-induced**

**protein conformations**

Andrew Stannard^1^*, Marc Mora^1^*, Amy E.M. Beedle^1^*, Marta Castro-López^1^, Stephanie Board^1^, Sergi Garcia-Manyes^1,2¶^

^1^Department of Physics, Randall Centre for Cell and Molecular Biophysics and London Centre for Nanotechnology, King’s College London, Strand, WC2R 2LS London, United Kingdom.

^2^The Francis Crick Institute, 1 Midland Road, London NW1 1AT, London, UK.

*These authors contributed equally to the work

^¶^Corresponding author: sergi.garcia-manyes@kcl.ac.uk

**Polyprotein engineering**

The Ig32 protein monomer was created by Thermofisher Scientific GenArt™ with incorporated BamHI, BglII and KpnI restriction sites and subcloned into pQE80L vector (Qiagen). (Ig32)_2_-PL-(Ig32)_2_ and (Ig27-SR73)_4_ polymers were engineered by restriction digest using the compatible cohesive end restriction enzymes BamHI and BglII between BamHI and KpnI sites. pFN18a talin R3 IVVI-Spy0128_2_ and PQE80L PL_8_ were kindly gifted from Julio Fernandez. PQE80L (Ig27-SR73)_4_, PQE80L PL_8_ and pQE80L Ig32_2_-PL-Ig32_2_ were subcloned into a modified pFN18a vector engineered with the AviTag™ (avidity) (sequence GLNDIFEAQKIEWHE). Constructs were cloned between the HaloTag at the N-terminus and the 6x histidine tag adjacent to the AviTag™ at the C-terminus. Recombinant plasmids were transformed in XL1Blue (Agilent Technologies) or Top10 (Thermofisher scientific) competent cells. Selected colonies were grown in Luria broth supplemented with 100 mg/ml ampicillin at 37 °C. Cells were lysed and plasmid DNA was purified before transfection using a Qiagen kit according to the manufacturer’s instructions.

Constructs were expressed in E. coli BLR(D3) cells (Novagen). Cells were grown in Luria broth supplemented with 100 mg/ml ampicillin at 37 °C. After reaching an OD_600_ of about 0.6, cultures were induced with 1 mM isopropyl-β-D-thiogalactopyranoside and grown at 25 °C for 16 h. Cells were resuspended in buffer 50 mM sodium phosphate (NaPi) pH 7.0, 300 mM NaCl, 10% glycerol and 1mM DTT supplemented with 100mg/ml lysozyme, 5 µg/ml DNase, 5 µg/ml RNase and 10mM MgCl_2_ and incubated on ice for 30 minutes. Cells were disrupted by a French press and lysate was centrifuged at 20,000 g for 40 minutes before purification by Talon affinity resin (Clontech) using wash buffer (50 mM NaPi pH 7.0, 300 mM NaCl, 10% glycerol, 1mM DTT, 20 mM imidazole). Protein was eluted with 250 mM imidazole. This was followed by gel filtration using a Superdex 200 10/300 GL column (GE Biosciences). Proteins were stored in gel filtration buffer 10 mM HEPES pH 7.2, 150 mM NaCl, 10% glycerol, 1 mM EDTA at -20°C.

Gel filtration fractions were pooled and concentrated using Amicon® filters with selected MWCO. Constructs were biotinylated using BirA Biotin Ligase (Avidity) following the suggested protocol (40 µM substrate, 1x buffer A, 1x buffer B, 2.5 µg enzyme BirA, at a final volume of 200 µl). The reaction was left for 3 hours at 30°C). Biotinylation was confirmed using Streptavidin HRP conjugate (Millipore) with biotinylated/unbiotinylated MBP-AviTag^TM^ fusion protein (Avidity) as controls.

**
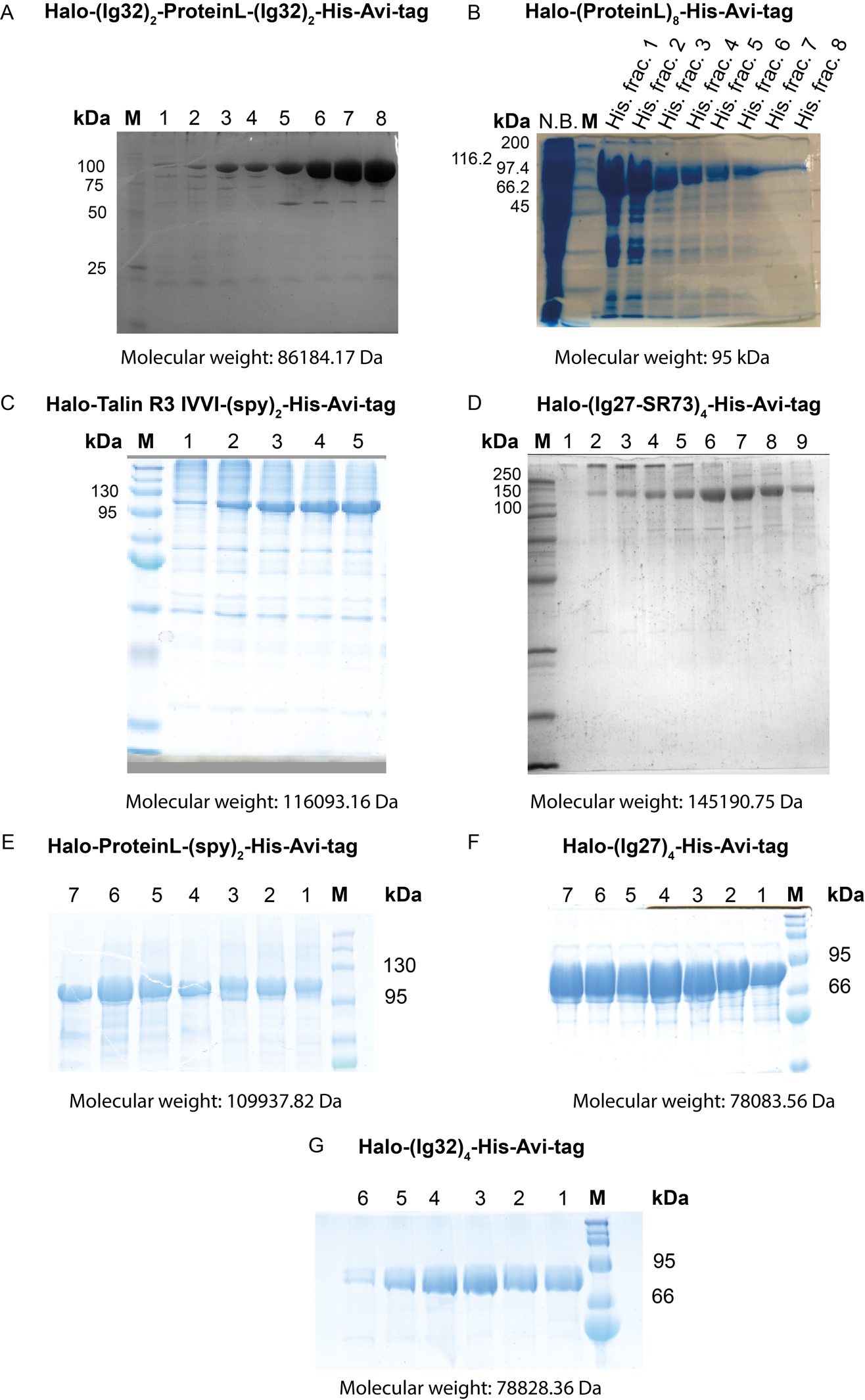
**

**SDS-Page gels of the different polyprotein constructs used throughout this work.**

**Single-molecule magnetic tweezers experiments**

Sample preparation and the single-molecule magnetic tweezers setup are based on those reported elsewhere^1^, with slight variations. Briefly, the single-molecule experiments were performed on a custom-made setup built on top of an inverted microscope (Nikon Eclipse TE2000-S) and using a 100x oil-immersion objective mounted on a high-precision positioner actuator (P-725 PIFOC objective scanner, Physik Instrumente). The sample was illuminated with white light (M590L3-C5, Thorlabs) and images were acquired using a CCD camera with 2 µs exposure time and a frame rate of $\sim$280 fps (Pike F-421, Allied Vision Technologies). The position of the permanent magnets (D33-N52, K&J Magnetics) was precisely controlled by a linear voice-coil (LFA-2010, Equipment Solutions) with 150 nm position resolution.

Single molecule experiments were performed in fluid chambers composed of two cover slides (40 × 24 mm and 22 × 22 mm, bottom and top respectively, thickness #1, Agar Scientific) with parafilm sandwiched in between. To construct these chambers, first, all cover slides were cleaned by sonication; with 1 % Hellmanex solution (60 °C for 30 minutes), then with acetone (R.T. for 30 minutes), and finally with ethanol (R.T. for 30 minutes), before drying at 100 °C for 30 minutes. In order to eliminate reflections from the magnetic faces located above the fluid chamber, the top surfaces of the top cover slides were coated with matt black spray paint (Rust-Oleum). The bottom cover slides were first activated by plasma cleaning (20 minutes) before silanization (immersion in 0.1% v/v 3-(aminopropyl)trimethoxysilane in methanol for 30 minutes). The silanized cover slides were then cured at 100 °C for 30 minutes. Fluid chambers were assembled by placing a parafilm cut-outs (40 × 24 mm with the central ~30 × 16 mm removed) between top and bottom cover slides followed by brief heating on a hot plate at 100 °C to ensure adhesion. After assembly, chambers were incubated with an aqueous glutaraldehyde solution (glutaraldehyde grade I 70 %; 0.01% v/v) for 60 minutes, followed by incubation with 0.02% w/v polystyrene beads/microspheres (~2.68 µm diameter, Spherotech). After 20 minutes, those beads unattached to chamber bottoms were washed out with PBS. Chambers were then incubated with a 10 µg/ml solution of the HaloTag amine (O4) ligand (Promega) in PBS, and left overnight in a humidity chamber. Finally, chambers were filled with a BSA-blocking buffer (20 mM Tris-HCL pH 7.3, 150 mM NaCl, 2 mM MgCl_2_ and 1% w/v sulfhydryl blocked BSA, MyBioSource), for a minimum of 12 hours before use. Once fluid chambers were ready, polyprotein constructs were freshly diluted in PBS buffer to a concentration ~0.01-0.02 nM and incubated in a chamber for 30 minutes to achieve HaloTag binding. Unreacted polyproteins were washed out with PBS. Finally, streptavidin-coated paramagnetic beads (Dynabeads M-270, Invitrogen) were added to the chamber to bind with the biotinylated polyprotein terminus for ~10 min. Unbound beads were then washed out with a 5 mM ascorbic acid solution in PBS (pH 7.3).

**Calculation of the stiffness of the magnetic bead potential**

Calculating the stiffness of the magnetic bead potential can be achieved with knowledge of the specifications of the magnetic beads and magnets used. The magnetic force on a paramagnetic bead is

$$\boldsymbol{F}=\nabla\left( \boldsymbol{m}\cdot\boldsymbol{B} \right)$$

where $\boldsymbol{m}$ is the magnetic moment of the bead and $\boldsymbol{B}$ is the magnetic flux density of the magnetic field; the spatial gradient of $\boldsymbol{F}$ gives the stiffness of the magnetic bead potential.

The magnetic moment of a paramagnetic bead will align to the field inducing the moment, with its magnitude depending on the field strength, i.e. $\boldsymbol{m}=m\left( B \right)\hat{\boldsymbol{B}}$. It has been shown^2, 3^ that the flux-density-dependence of the magnetic moment of an M-280 Dynabead (as used in our experiments) can be approximated by the Langevin function

$$m\left( B \right)=m_{S}\left[ \coth\left( B/{B_{0}} \right)-{B_{0}}/B \right]$$

where $m_{S}=1.66\times{10}^{-13}\text{A}\text{m}^{2}$ is the saturation magnetic moment and $B_{0}=15.5 \text{mT}$ a characteristic flux density, see Fig. S1A. At low flux densities, $B\ll B_{0}$, these beads are superparamagnetic; their magnetisation saturates at high flux densities, $B\gg B_{0}$.

Our magnetic tweezers set-up uses neodymium-based cylindrical magnets (D33-N52, K&J Magnetics), specified by radius $R=2.38 \text{mm}$, length $L=4.76 \text{mm}$, and surface flux density $B_{\text{S}}=662 \text{mT}$; the latter corresponds to a magnetisation of $M=1.18\times{10}^{6}\text{A}\text{m}^{\text{-1}} \left( ={2B_{\text{S}}\left( R^{2}+L^{2} \right)^{1/2}}/{\mu_{0}L} \right)$. 6 of these magnets are used in total: 3 magnets are aligned magnetically-parallel in face-to-face contact (to effectively make one magnet of length $L=14.3 \text{mm}$); two of these stacks are aligned magnetically-antiparallel in side-to-side contact. In cylindrical coordinates, the azimuthally-symmetric magnetic scalar potential outside a cylindrical magnetic is

$$\psi\left( \rho,z \right)=\frac{M}{4\pi}\int_{0}^{L} \int_{0}^{2\pi} \int_{0}^{R} \frac{r\left( z+l \right)}{\left( \rho^{2}-2\rho r\cos\theta+r^{2}+\left( z+l \right)^{2} \right)^{3/2}}drd\theta dl$$

with $z=0$ corresponding to one of the magnetic faces. From the scalar potential, the magnetic flux density can be found by

$$\boldsymbol{B}=-\mu_{0}\nabla\psi$$

where $\mu_{0}=1.26\times{10}^{-6} \text{H/m}$ is the permeability of free space. Fig. S1B shows the calculation of the magnetic flux density in the plane that transects both magnet stacks.

Fig. S1C showing the corresponding paramagnetic force on an M-280 Dynabead in the vicinity of the magnet stacks. Measurements are performed on magnetic beads located on the axis defined by the side-to-side/stack-to-stack contact line, with positioning achieved through lateral movement of the bead-containing sample. The magnet-bead separation, $z$, the perpendicular distance between the bead and the magnetic faces, is controlled by vertical positioning of the magnets. Fig. S1D shows the paramagnetic force as a function of magnet-bead separation along the central axis.

The stiffness of the potential experienced by the paramagnetic bead is simply given by the gradient of this force, shown in Fig. S1E over two decades of force, the typical force range used in magnetic tweezers experiments. Also shown is an estimation of the stiffness of a typical protein construct tethered to a bead in our experiments. The force-dependent stiffness of a freely-jointed chain of contour length $L$ is

$$k\left( F \right)=\left( {k_{B}T}/{bL} \right)\left[ 1-\coth^{2} \left( {bF}/{k_{B}T} \right)-\left( {k_{B}T}/{bF} \right)^{2} \right]^{-1}$$

where $k_{B}T=4.04 \text{pN nm}$ is thermal energy at room temperature, $b=1.1 \text{nm}$ is the Kuhn length we use for protein constructs, and $L=100 \text{nm}$ is the order of magnitude of contour length of constructs used throughout here. Over this force range the stiffness of the protein far exceeds that of the magnetic bead potential (by 5 or more orders of magnitude), and thus the ‘trap’ stiffness experienced by magnetic beads in our magnetic tweezers experiments is completely dominated by the protein stiffness, and thus our measurements of ‘magnetic trap’ stiffness are indeed measurements of protein stiffness. By comparison, in the force range of interest here, protein stiffness is at least an order of magnitude less than a typical cantilever using for SMFS AFM (10 pN/nm), where the stiffness of the probe-protein coupled system is dominated by the probe.

Fig. S1E also illustrates the insignificant change in paramagnetic force when a protein (that a bead is tethered to) folds/unfolds to lengthen/shorten the magnet-bead separation. For example, at a typical force of ~10 pN, the stiffness of the magnetic bead potential is ~10^-5^ pN/nm. At this force, the change in average bead position due to a protein L folding/unfolding event is ~10 nm, meaning ~10^-4^ pN change in force, i.e. only one part in ten thousand, thus insignificant.


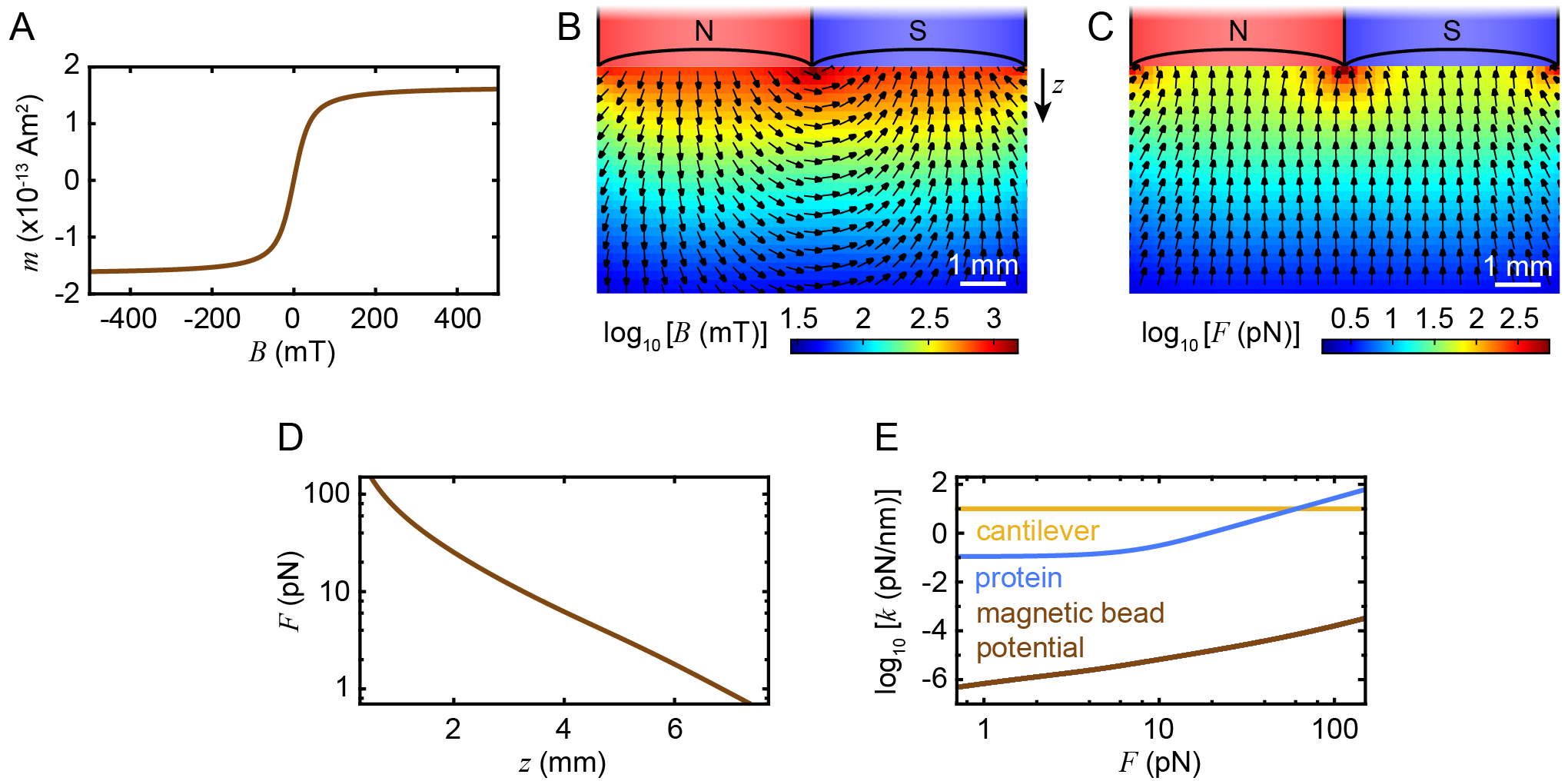


**Figure S1. Single molecule magnetic tweezers experiments allow measurement of the probe-independent stiffness of a protein under force. (A)** Estimation of the magnetic moment of an M-280 Dynabead as a function of magnetic flux density. **(B)** Magnetic flux density in the vicinity of the cylindrical magnets used in our experimental set-up. **(C)** Combining (A) and (B) gives the position-dependent force on a magnetic bead. **(D)** The vertical force along the axis defined by the side-to-side contact between cylindrical magnet stacks. **(E)** Comparing the force-dependent stiffnesses due to the magnetic potential to an approximation to a typical protein construct clearly show the former to be insignificant, and thus the stiffness of the ‘magnetic trap’ is in fact the protein stiffness. Also shown is the stiffness of a typical cantilever used in SMFS AFM experiments, for comparison.

**Compliance of chain models under a constant force constraint**

The equipartition theorem applies to harmonic oscillators, such as classical Hookean springs of linear elasticity. In thermal equilibrium, the mean potential energy of a spring of constant stiffness $k$ is

$$\left\langle U \right\rangle={k\sigma^{2}}/2={k_{B}T}/2$$

where $\sigma^{2}=\left\langle x^{2} \right\rangle-\left\langle x \right\rangle^{2}$ is the mean squared displacement from the mean equilibrium extension $\left\langle x \right\rangle$ and $k_{B}T$ is the thermal energy. Using thermodynamic beta $\beta=\left( k_{B}T \right)^{-1}$ for convenience, the above equation rearranges to a simple expression for compliance, $c=k^{-1}$, of a classical spring in relation to fluctuations in its extension

$$c=\beta\sigma^{2}$$

It is well known that (bio)polymers, and the chain models that can be used to describe them, exhibit nonlinear elasticity – a force-dependent compliance. As such, it is not immediately obvious that the simple relation above still holds for proteins under tensile force. For a chain model under a constant force constraint, $F$, the energy associated with microstate $\nu$, $E_{\nu}$, is the sum of the internal energy of the configuration, $U_{\nu}$, and the potential energy under the constraining force, $-Fx_{\nu}$, where $x_{\nu}$ is the extension (end-to-end distance)

$$E_{\nu}=U_{\nu}-Fx_{\nu}$$

Different models have different forms of $U_{\nu}$: ideal chains (e.g. freely-jointed chains) are special cases in which there are no internal interactions, thus $U_{\nu}=0$ for all $\nu$; for Hookean springs $U_{\nu}={kx_{\nu}^{2}}/2$; for a worm-like chains $U_{\nu}=\left( {pk_{B}T}/2 \right)\int\left[ {\partial^{2}\boldsymbol{r}_{\nu}\left( s \right)}/{\partial s^{2}} \right]^{2}ds$ where $p$ is the persistence length and $\boldsymbol{r}_{\nu}\left( s \right)$ is the position vector along the chain coordinate for microstate $\nu$. In the isothermal-isoforce ensemble of these chain models, the probability of microstate $\nu$ is $p_{\nu}=Z^{-1}e^{-\beta E_{\nu}}$, where the partition function is the sum of Boltzmann factors over all microstates

$$Z=\sum_{\nu} e^{-\beta E_{\nu}}$$

from which the mean extension can be found as

$$\left\langle x \right\rangle=\sum_{\nu} p_{\nu}x_{\nu}=Z^{-1}\sum_{\nu} x_{\nu}e^{-\beta E_{\nu}}$$

Since the mean extension of a chain model is (typically) a nonlinear function of force, its compliance (i.e. derivative of mean extension with respect to force) will (typically) be force-dependent, and given by

$$c\equiv\frac{d\left\langle x \right\rangle}{dF}=\beta\left[ Z^{-2}\sum_{\nu} \frac{dE_{\nu}}{dF}e^{-\beta E_{\nu}}\sum_{\nu} x_{\nu}e^{\beta Fx_{\nu}}-Z^{-1}\sum_{\nu} x_{\nu}\frac{dE_{\nu}}{dF}e^{-\beta E_{\nu}} \right]$$

Since the internal energy of a microstate is solely dependent on the microstate configuration, ${dE_{\nu}}/{dF}=-x_{\nu}$ for all chain models. This simplifies the above expression to

$$c\equiv\frac{d\left\langle x \right\rangle}{dF}=\beta\left[ Z^{-1}\sum_{\nu} x_{\nu}^{2}e^{-\beta E_{\nu}}-\left( Z^{-1}\sum_{\nu} x_{\nu}e^{\beta Fx_{\nu}} \right)^{2} \right]$$

This expression can be further simplified using expressions for the mean extension (above), the mean squared extension

$$\left\langle x^{2} \right\rangle=\sum_{\nu} p_{\nu}x_{\nu}^{2}=Z^{-1}\sum_{\nu} x_{\nu}^{2}e^{\beta Fx_{\nu}}$$

to give

$$c=\beta\left[ \left\langle x^{2} \right\rangle-\left\langle x \right\rangle^{2} \right]=\beta\sigma^{2}$$

This shows that the compliance expression as given by the equipartition theorem applied to a spring of linear elasticity is also valid for any chain model under a constant force constraint.

**Compliance calculation from the freely-jointed chain (FJC) model of polymer elasticity**

The freely-jointed chain (FJC) model is a facile, yet powerful, way to describe the mechanical behaviour of polymers, including proteins, and is the simplest ideal chain model. An FJC consists of $n$ rigid segments of fixed length, $b$ – the Kuhn length – connected via free joints (no steric hinderance/restrictions in either radial or azimuthal angles between adjacent segments). Each of these Kuhn segments represents a section of polymer that can be approximated to be behaving as an independent, discrete unit. The contour length (maximum end-to-end separation) of an FJC is simply given by $L=nb$, and it is a well-known result that the partition function of an FJC under the constraint of a constant tensile force $F$ is

$$Z=\left[ 4\pi\left( \beta bF \right)^{-1}\sinh\left( \beta bF \right) \right]^{n}$$

Above we showed in the previous section that $\partial_{F}Z=\beta Z\left\langle x \right\rangle$ for any ideal chain, thus

$$\left\langle x \right\rangle=\left( \beta Z \right)^{-1}\partial_{F}Z=\beta^{-1}\partial_{F}\left( \ln Z \right)$$

and for the FJC model the force-dependent average extension is

$$\left\langle x \right\rangle=\beta^{-1}n\partial_{F}\left\{ \ln\left[ 4\pi\left( \beta bF \right)^{-1}\sinh\left( \beta bF \right) \right] \right\}=L\left[ \coth\left( \beta bF \right)-\left( \beta bF \right)^{-1} \right]$$

where the function in squared brackets can be recognised as the Langevin function of the dimensionless force $\beta bF$.

Since $c\equiv\partial_{F}\left\langle x \right\rangle$, an expression for the force-dependent compliance of an FJC is then

$$c=\beta bL\left[ 1-\coth^{2} \left( \beta bF \right)+\left( \beta bF \right)^{-2} \right]$$

It is relatively trivial, but time-consuming, to show that this same result can be obtained by using $c=\beta\sigma^{2}=\beta\left\langle x^{2} \right\rangle-\beta\left\langle x \right\rangle^{2}$ after explicitly finding $\left\langle x \right\rangle=\left( \beta Z \right)^{-1}\partial_{F}Z$ and $\left\langle x^{2} \right\rangle=\beta^{-2}Z^{-1}\partial_{F}^{2}Z$ from the FJC partition function.

The process of protein unfolding is associated with an increment in contour length of $\Delta L$, and thus, according to the FJC model, is also associated with changes in average extension and compliance of

$$\Delta\left\langle x \right\rangle=\Delta L\left[ \coth\left( \beta bF \right)-\left( \beta bF \right)^{-1} \right]$$

and

$$\Delta c=\beta b\Delta L\left[ 1-\coth^{2} \left( \beta bF \right)+\left( \beta bF \right)^{-2} \right]$$

respectively.

_
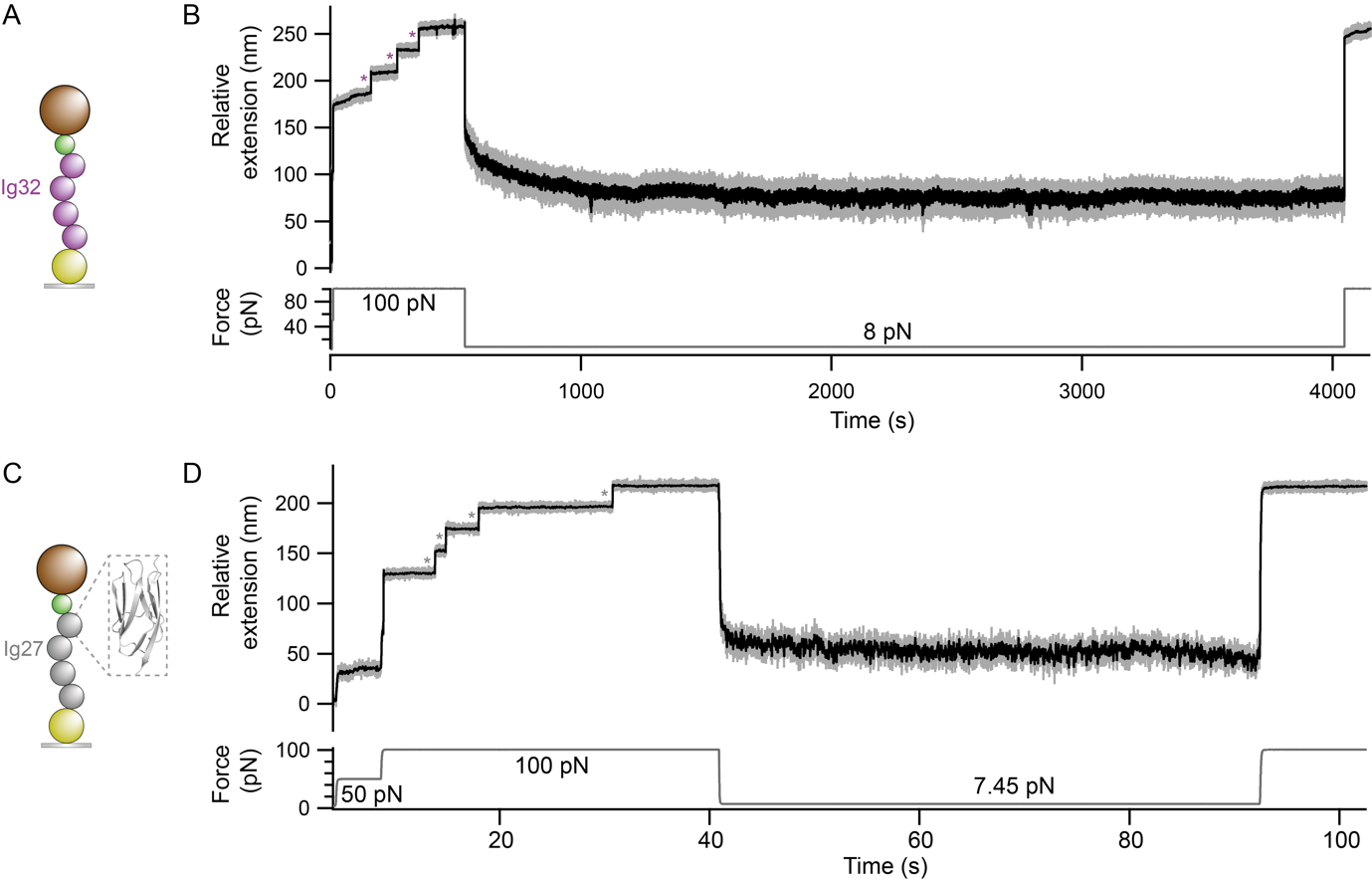
_

**Figure S2. Inactivity of titin domains at low forces.** **(A)** Illustration of the (Ig32)_4_ construct being pulled in the magnetic tweezers instrument. **(B)** (Top) Raw (grey) extension-time measurement with smoothed (black) overlay. (Bottom) Corresponding force-time protocol. Due to the high mechanical stability of Ig32, mechanical unfolding only occurs after exposure to high force (100 pN) for several minutes, hallmarked by $\sim$25 nm step-wise increase of the polyprotein chain (starred). Following the same experimental protocol employed to study the folding dynamics of protein L, the force was then quenched down to 8 pN. During this period, the previously unfolded Ig32 domains remain unfolded for one hour, further confirmed by the absence of extension steps when high force is reapplied. **(C)** Illustration of the (Ig27)_4_ construct pulled at constant force with magnetic tweezers. **(D)** (Top) Raw (grey) extension-time measurement with smoothed (black) overlay, accompanied with the corresponding force protocol (bottom). Due to the high mechanical stability of Ig27 (albeit less than Ig32), mechanical unfolding only occurs after exposure to high force (100 pN) for 30 seconds, hallmarked by a $\sim$22 nm step-wise increase in protein extension, corresponding to the stochastic unfolding of each of the protein domains within the polyprotein chain. Similar to the case of Ig32, when force is quenched down to 7.45 pN, the individual Ig27 domains remain unfolded for 30 seconds (the timescale of the nesprin folding trajectories), confirmed by the absence of extension steps when high force is reapplied. These data show that both Ig32 and Ig27 are inactive when exposed to the forces for the corresponding durations that they experience when part of Ig32_2_-PL-Ig32_2_ and (Ig27-SR73)_4_ constructs, respectively.

**Variance measurement from raw single molecule data. Data processing.**

During an experiment, the positions of i) the magnetic bead tethered to the protein-construct-of-interest and ii) a nearby surface-bound reference (non-magnetic) bead are recorded, see Figs. S3A&B, respectively, for data acquired using the protein L monomer construct, centred around an unfolding event. The same low-frequency thermal drift dominates both signals, disguising the unfolding event. Both signals also display high-frequency fluctuations – clearly larger in the magnetic bead signal. The main purpose of recording the reference bead position is to track the thermal drift of the experimental apparatus, enabling drift correction of the magnetic bead recording. The reference bead position is subject to measurement uncertainty, and so the reference signal is low-pass filtered to provide the drift correction. A Gaussian-weighted moving average filter (Gaussian smoothing) is used; the filter window width is optimized to discriminate between the high-frequency measurement uncertainty and the low-frequency thermal drift (a ~1 s window is typically optimal), see Fig. S3C. This low-frequency component is then subtracted from the magnetic bead position, see Fig. S3D, to give the relative extension of the protein-construct-of-interest, $x$ (now clearly displaying the domain unfolding event). Here, the change in average extension due to unfolding, $\Delta\left\langle x \right\rangle$, is calculated as the difference in average relative extension one second either side of the event (or less if another event occurs within one second). From the relative extension, the extension variance, $\sigma^{2}=\left\langle x^{2} \right\rangle-\left\langle x \right\rangle^{2}$, can be found, Fig. S3E shows the (moving) variance, where it is clear that upon domain unfolding there is a variance increase.

This measured variance, $\sigma_{M}^{2}$, has two components: the variance due to the true extension fluctuations of the protein-construct-of-interest (which in itself has two components: the protein-of-interest; and the rest of the construct); and the variance due to position measurement uncertainty. This second, unwanted, contribution will be the same for both the magnetic and reference bead. Fig. S3E also shows the (moving) variance of the high-frequency residual of the reference bead – the position measurement uncertainty variance, $\sigma_{R}^{2}$. With this knowledge, one can isolate the variance solely due to true extension fluctuations, $\sigma_{M}^{2}-\sigma_{R}^{2}$, which can be used to calculate the compliance of the protein-construct-of-interest, $c=\left( \sigma_{M}^{2}-\sigma_{R}^{2} \right)/{k_{B}T}$, see Fig. S3F.

It should be noted that although here we are calculating an absolute compliance, these absolute values will always correspond to the polyprotein construct as a whole, and never to just the protein-of-interest. As such, even with these absolute values, only differences in compliance can meaningfully correlate to the un/folding of the protein-of-interest (when the rest of the construct stays the same). Therefore, when combining experimental data, we only ever combine differences in compliance – these compliance differences will naturally be the same whether calculated from absolute compliance (calculated as described above) or relative compliance (i.e. calculated without subtracting the position measurement uncertainty).

The power of these difference measurements means that the compliance of the rest of the construct is irrelevant. This allows for versatility in heteropolyprotein design, giving the freedom to use a variety of ‘inextensible’ marker proteins (e.g. Ig27, Ig32, and Spy0128 are used here), with the position of the protein(s)-of-interest within these constructs being inconsequential. The independence of compliance difference on the rest of the construct is also particularly important in relation to the fact that the HaloTag is sometimes observed to unfold – this will significantly increase the absolute compliance of the construct. However, differences in compliance when a protein-of-interest un/folds will be independent of whether the HaloTag is unfolded or not, and so results of different constructs can be combined irrespective of the HaloTag state.


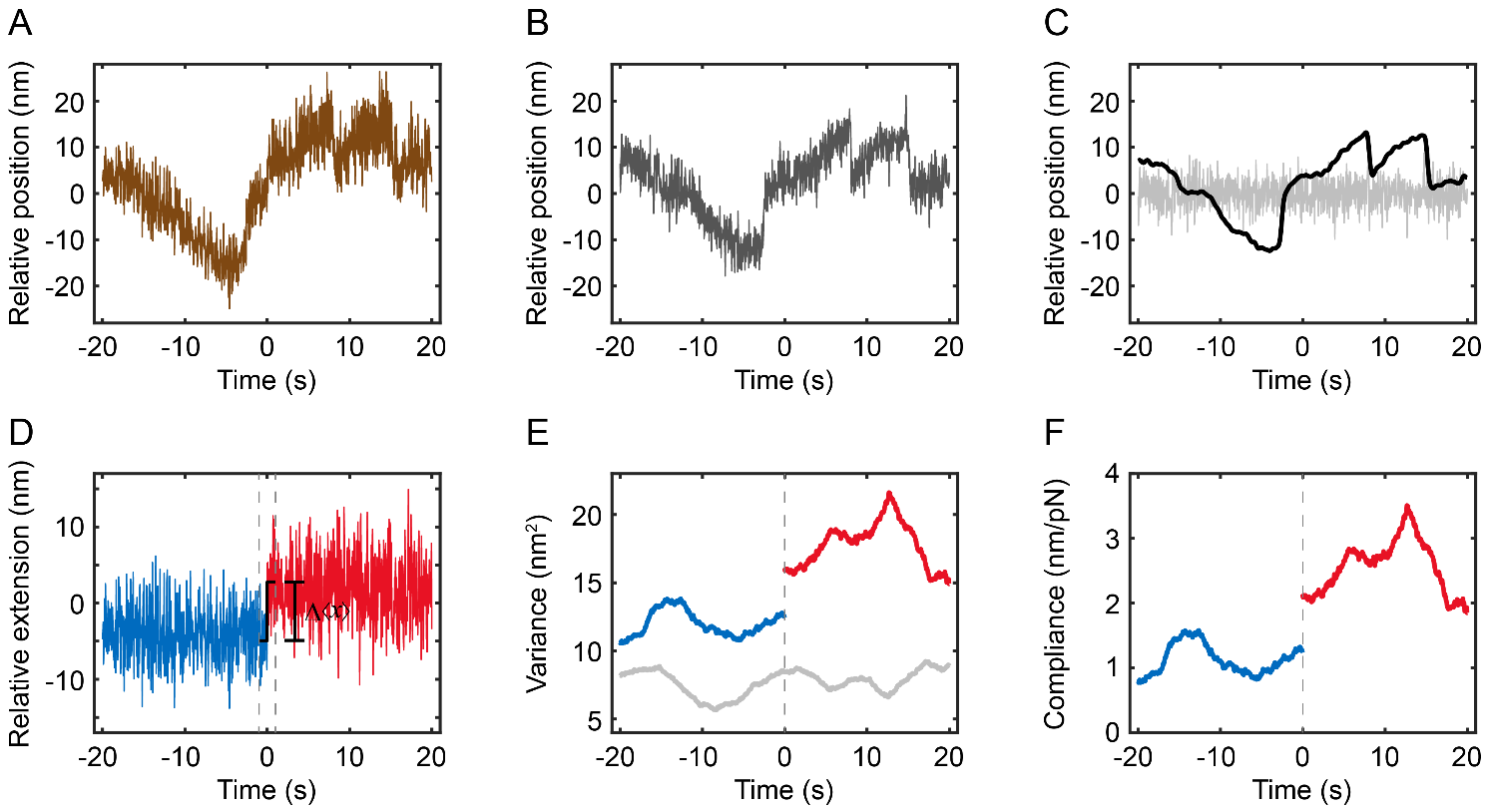


**Figure S3.** Relative positions of **(A)** a magnetic bead tethered to a protein-construct-of-interest and **(B)** a nearby surface-bound reference bead, with a domain unfolding event occurring at time zero. **(C)** Reference bead signal separated into low- (black) and high-frequency (light grey) components using a Gaussian-weighted moving average filter. **(D)** Relative extension of the protein-construct-of-interest, highlighting the folded (blue) and unfolded (red) domain states, the difference in average position one second either side of the unfolding event is the change in average extension. **(E)** Moving variance (three second window, or less if within three seconds of the event) of the relative extension, showing a clear distinction between the folded (blue) and unfolded (red) domain state. Also shown is the moving variance (3 s window) of the high-frequency component of the reference bead position (light grey). **(F)** Compliance of the protein-construct-of-interest before (blue) and after (red) domain unfolding, showing a clear increase in compliance accompanying domain unfolding.

**
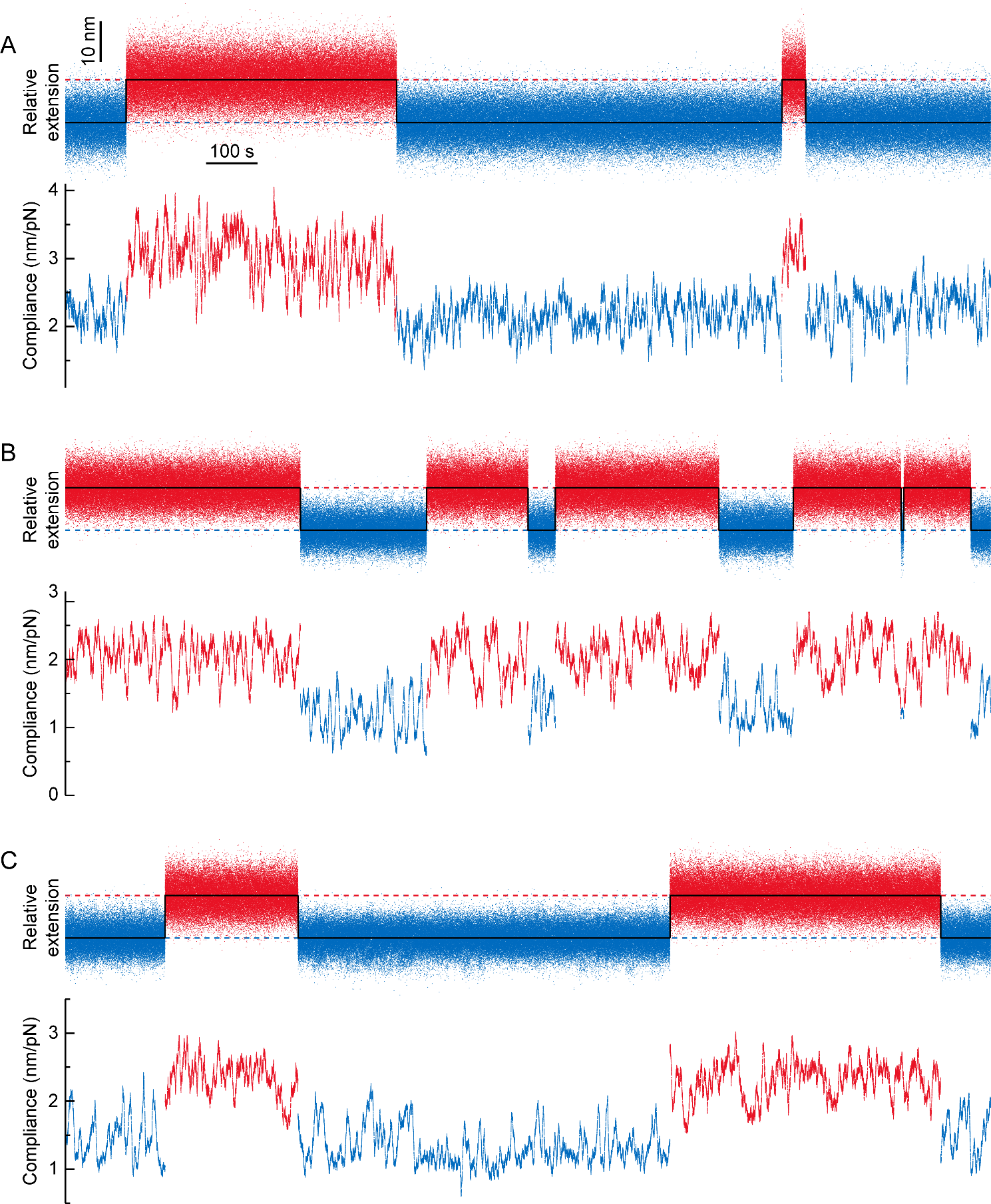
**

**Figure S4.** **Three further representative examples of protein L hopping between folded and unfolded states at 8.1 pN**. These measurements provide extension and compliance changes respectively of **(A)** Δ<*x*> = 10.5 ± 0.9 nm and Δ*c* = 0.89 ± 0.03 nm/pN, **(B)** Δ<*x*> = 10.4 ± 0.3 nm and Δ*c* = 0.83 ± 0.07 nm/pN, and **(C)** Δ<*x*> = 10.4 ± 0.6 nm and Δ*c* = 0.82 ± 0.12 nm/pN.


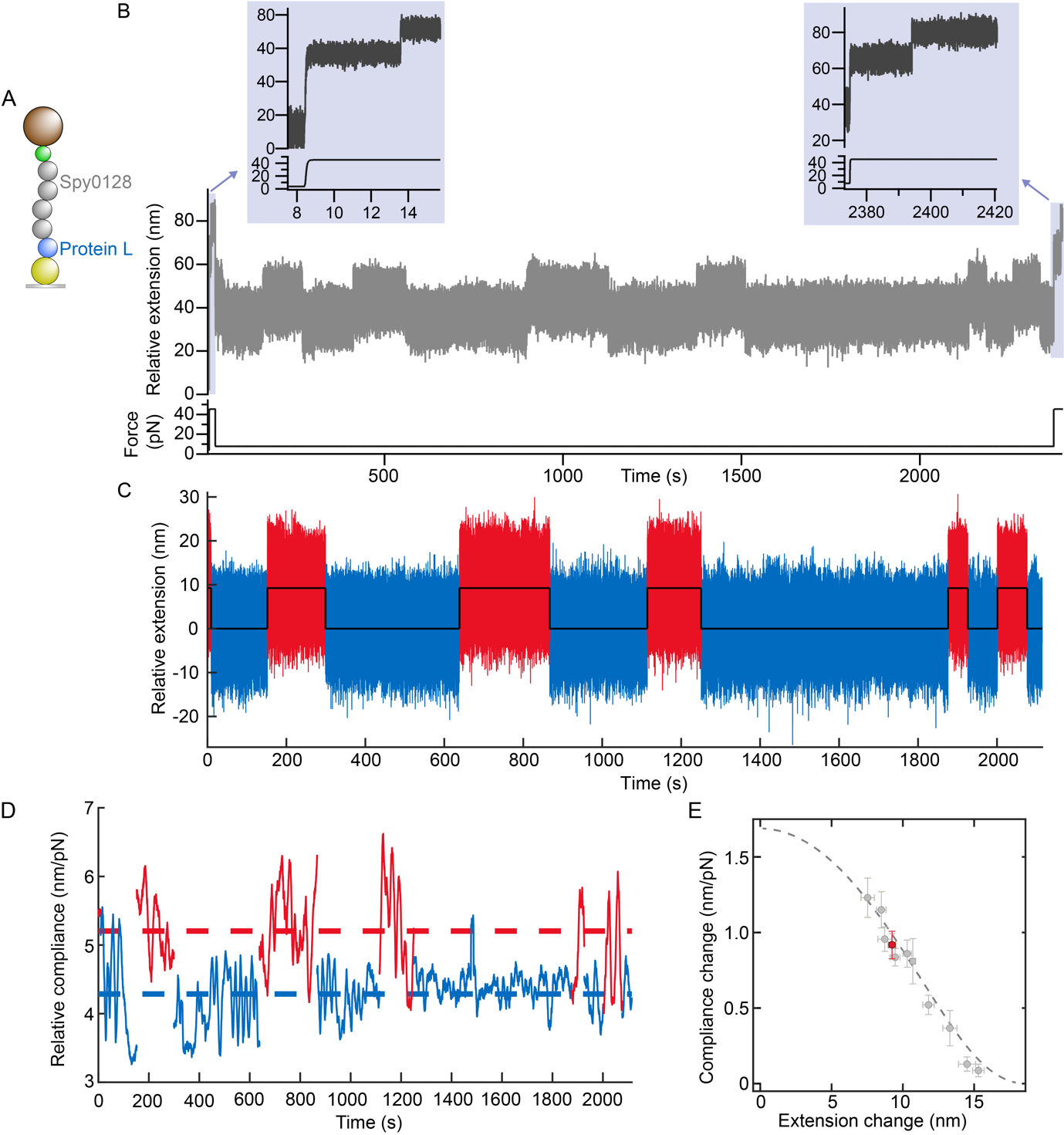


**Figure S5. The folding dynamics of protein L is independent of the rest of the polyprotein construct. (A)** Schematic illustration of the [PL-(Spy0128)_2_] construct. **(B)** Raw extension-time measurement of the protein L monomer hopping between the folded and unfolded states. The force-time trajectory informs on the employed force protocol. The construct is initially pulled at 45 pN, which extends and unfolds the PL monomer as hallmarked by the $\sim$15 nm step (left zoom). After 17 s, the force is quenched down to 7.9 pN for 2350 s. During this time the PL monomer hops between the folded (slightly favoured) and unfolded states in steps of Δ<*x*> = 9.2 ± 0.2 nm. A final pulse at 45 pN triggers the (re)unfolding of the PL monomer (right zoom). **(C)** Protein extension data during the 7.9 pN pulse clearly highlighting 11 individual (un)folding transitions. **(D)** Relative compliance of (C) (calculated over a 10-second moving window), showing step-wise changes (Δ*c* = 0.92 ± 0.09 nm/pN) concomitant to the PL individual unfolding and refolding events. **(E)** A modified version of Fig. 4D from the manuscript, with an additional data point using the values from (C) and (D). This shows that the behaviour of protein L in the [PL-(Spy0128)_2_] construct (red) and the [Ig32_2_-PL-Ig32_2_] construct (grey) are essentially identical, and that our results are independent of the rest of the construct.

**
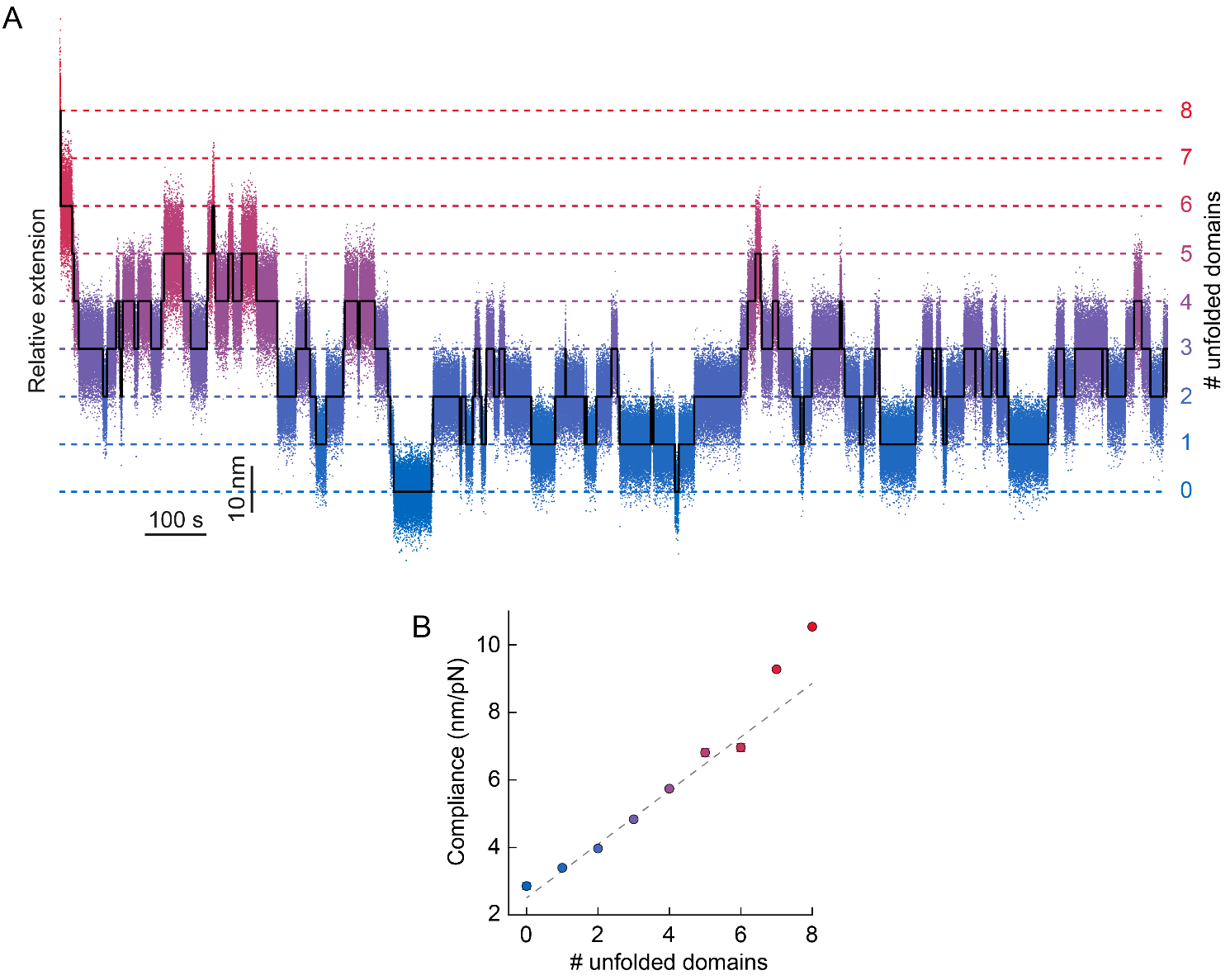
**

**Figure S6. (A)** A further representative example of a protein L octamer hopping between different levels characterising different numbers of un/folded domains at 8.1 pN. 123 transitions are observed, with an average change in extension of Δ<*x*> = 10.2 ± 0.1 nm. **(B)** A linear fit to compliance against the number of unfolded domains yields a gradient which gives Δ*c*/domain = 0.79 ± 0.10 nm/pN.

**
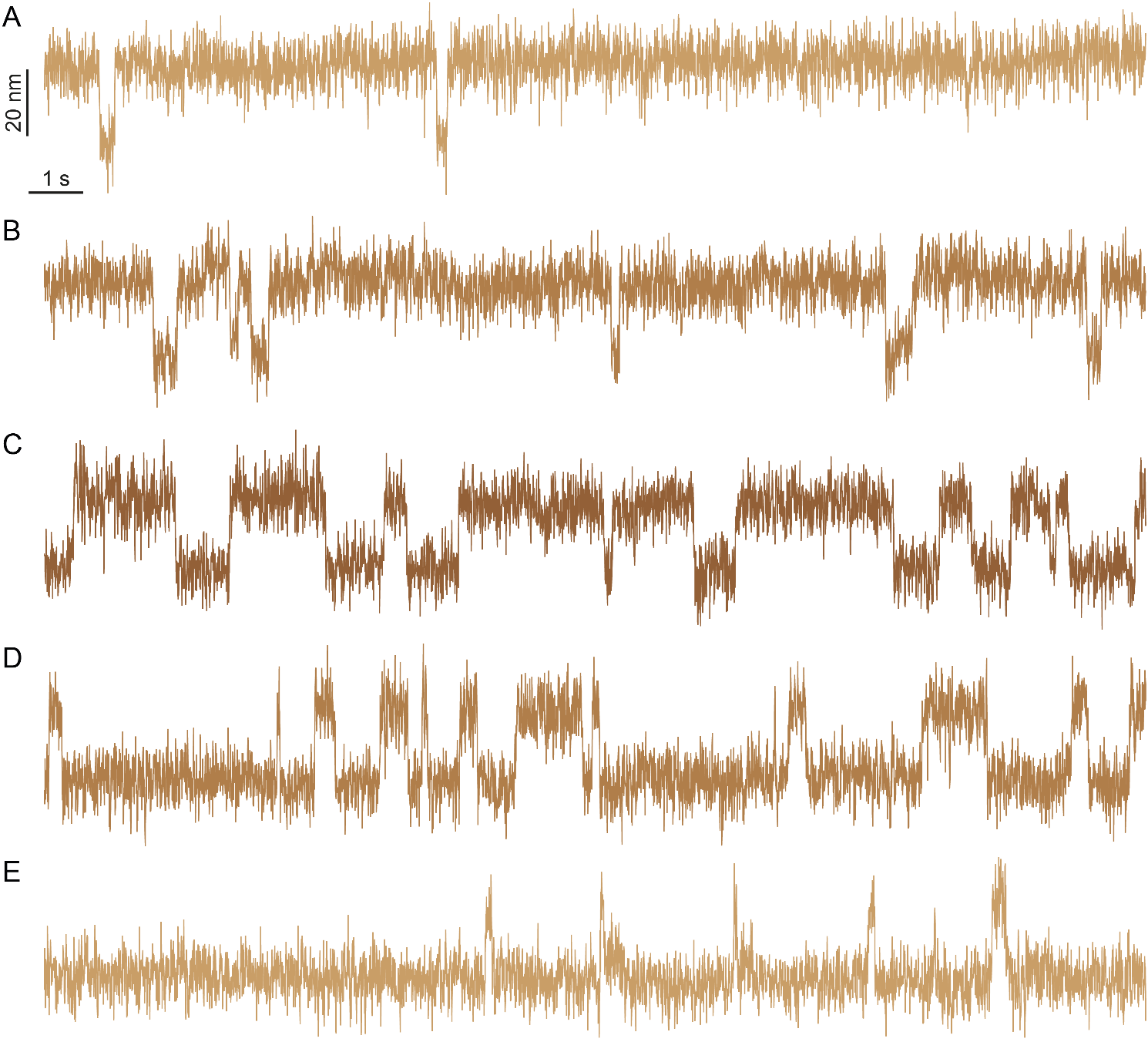
**

**Figure S7. Force dependency of talin R3 IVVI folding under force.** Several measurements of relative extension against time for the talin R3 IVVI construct held at **(A)** 8.9 pN, **(B)** 8.1 pN, **(C)** 7.8 pN, **(D)** 7.4 pN, and **(E)** 6.9 pN. At forces above [below] that which provides equal occupation of folded and unfolded states ⎯ (C) here⎯ , the folded [unfolded] state exists too briefly to reliable make variance (and therefore compliance) measurements within the bandwidth (~280 Hz) of our instrumentation. Critically, the bandwidth of our experiments might be insufficient to capture the putative transient intermediate states proposed for the talin R3 subdomain^4^.

**
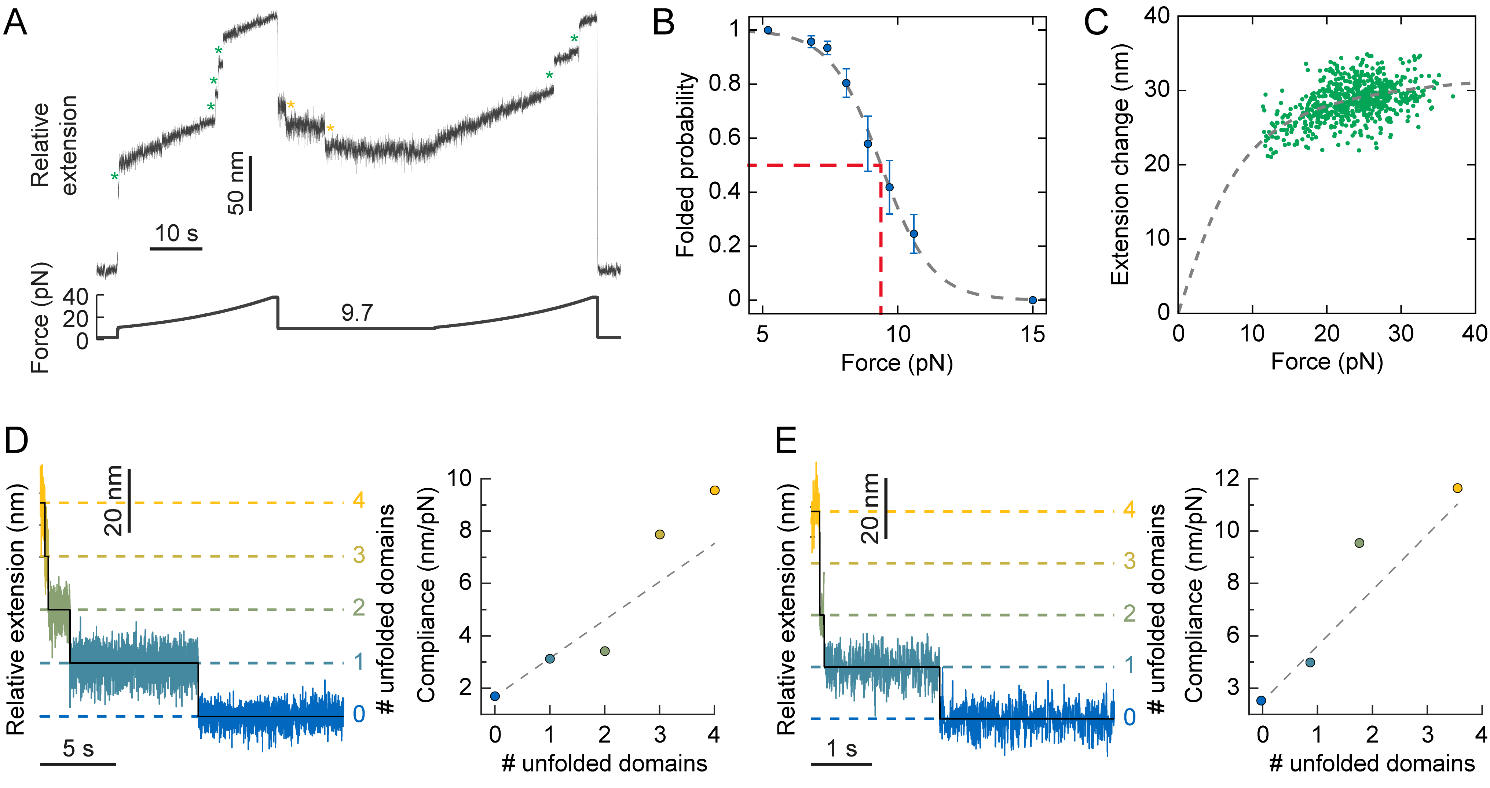
**

**Figure S8. Further examples of nesprin folding characterization (A)** A representative example of the force protocol used to determine the force-dependent folded probability of an SR73 domain. Initial ramping of force up to 38 pN reveals 4 unfolding events, force is then quenched to 9.7 pN for 30 seconds. A second ramping of force up to 38 pN only reveals 2 unfolding events. Consequently, this particular trajectory suggests the folded probability at this force is 2/4 = 0.5 i.e. two out of four possible domains were folded when high force was reapplied. For any given force, this procedure is repeated many times to find an average. Only trajectories featuring a minimum of three, and a maximum of four unfolding events in the initial force ramp were considered for analysis. Standard error for the folded probability was estimated through the bootstrap method, where each individual unfolding was treated as a completely separate event. **(B)** Repeating this procedure for a range of quench forces allows folded probability against force to be plot, and subsequently fit to a sigmodal of the form $1/\left( 1+\exp\left( \left( F-F_{0.5} \right)/\sigma\right) \right)$where the equilibrium hopping force of SR73 is found to be *F_0.5_* = 9.4 ± 0.2 pN (and *σ* = 0.94 ± 0.16 pN). **(C)** To estimate the contour length increment, the force and associated extension change values of all 703 unfolding events observed during force ramp measurements, as described in (A), were plotted and fit to the FJC model of force-dependent extension change $\Delta L\left[ \coth\left( \beta bF \right)-\left( \beta bF \right)^{-1} \right]$ with *b* = 1.1 nm and β = 1/4.04 = 0.248 pN^-1^ nm^-1^ to yield Δ*L* = 34.2 ± 0.2 nm. **(D, E)** Two further representative examples of the SR73 tetramer folding at 7.4 pN. When analysed these measurements provide extension and compliance changes respectively of (D) Δ<*x*> = 16.7 ± 1.4 nm and Δ*c*/domain = 1.46 ± 0.43 nm/pN and (E) Δ<*x*> = 17.1 ± 0.7 nm and Δ*c*/domain = 1.90 ± 0.84 nm/pN.

**References**

1. Popa, I.; Rivas-Pardo, J. A.; Eckels, E. C.; Echelman, D. J.; Badilla, C. L.; Valle-Orero, J.; Fernandez, J. M., A HaloTag Anchored Ruler for Week-Long Studies of Protein Dynamics. *J Am Chem Soc* **2016,** *138* (33), 10546-53.

2. Fonnum, G.; Johansson, C.; Molteberg, A.; Mørup, S.; Aksnes, E., Characterisation of Dynabeads® by magnetization measurements and Mössbauer spectroscopy. *Journal of Magnetism and Magnetic Materials* **2005,** *293* (1), 41-47.

3. Lipfert, J.; Wiggin, M.; Kerssemakers, J. W.; Pedaci, F.; Dekker, N. H., Freely orbiting magnetic tweezers to directly monitor changes in the twist of nucleic acids. *Nat Commun* **2011,** *2*, 439.

4. Mykuliak, V. V.; Haining, A. W. M.; von Essen, M.; Del Rio Hernandez, A.; Hytonen, V. P., Mechanical unfolding reveals stable 3-helix intermediates in talin and alpha-catenin. *PLoS Comput Biol* **2018,** *14* (4), e1006126.
